# Effects of Liqi Tongbian decoction on gut microbiota, SCFAs production, and 5-HT pathway in STC rats with Qi Stagnation Pattern

**DOI:** 10.3389/fmicb.2024.1337078

**Published:** 2024-03-15

**Authors:** Qihong Liu, Danfeng Ke, Youqin Chen, Aling Shen, Liya Liu, Lunan Hu, Yan Ren, Wenyi Fang, Peilin Zhao, Thomas J. Sferraf, Yunfeng Luo, Xiao Ke

**Affiliations:** ^1^Department of Gastroenterology, The Second People's Hospital Affiliated to Fujian University of Traditional Chinese Medicine, Fuzhou, China; ^2^Department of Pediatrics, Case Western Reserve University School of Medicine, Rainbow Babies and Children's Hospital, Cleveland, OH, United States; ^3^Academy of Integrative Medicine, Fujian University of Traditional Chinese Medicine, Fuzhou, China

**Keywords:** slow transit constipation, Liqi Tongbian decoction, gut microbiota, short-chain fatty acids, serotonin

## Abstract

Slow transit constipation (STC) is a common and debilitating condition characterized by delayed colonic transit and difficulty in fecal expulsion, significantly impacting patients’ physical and mental wellbeing as well as their overall quality of life. This study investigates the therapeutic potential of Liqi Tongbian Decoction (LTD) in the treatment of STC, especially in cases involving the context of Qi stagnation, through a multifaceted approach involving the modulation of intestinal flora and short-chain fatty acids (SCFAs). We employed a rat model of STC with Qi Stagnation Pattern, established using the “loperamide + tail-clamping provocation method,” to explore the effects of LTD on fecal characteristics, intestinal motility, and colonic pathology. Importantly, LTD exhibited the ability to increase the richness, diversity, and homogeneity of intestinal flora while also modulating the composition of microorganisms. It significantly increased the production of SCFAs, especially butyric acid. Moreover, LTD exerted a substantial influence on the synthesis of serotonin (5-HT) by modulating the expression of tryptophan hydroxylase (TPH) and interacting with the 5-HT4 receptor (5-HT4R), resulting in enhanced colonic motility. Correlation analyses revealed a positive correlation between certain bacterial genera, such as Lachnospiraceae_NK4A136 spp. and Clostridiales spp. and the concentrations of butyric acid and 5-HT. These results suggest a mechanistic link between microbiome composition, SCFAs production, and 5-HT synthesis. These findings highlight the potential of LTD to alleviate STC by facilitating a beneficial interplay among intestinal flora, SCFAs production, and 5-HT-mediated colonic motility, providing novel insights into the management of STC with Qi Stagnation Pattern.

## Introduction

Slow transit constipation (STC) is a common subtype of functional constipation (FC), primarily characterized by reduced colonic transit capacity and decelerated transit speed. Clinically, it is characterized by infrequent bowel movements (>3 days/times; more than 3 days between movements?) and dry feces (Bharucha and Lacy, 2020). The incidence of STC is increasing due to society’s aging population and changes in dietary and lifestyle habits (Wang et al., 2022). Epidemiological data reveal that FC affects up to 15.3% of adults, and STC accounts for approximately 55% of FC (Barberio et al., 2021). STC is universally recognized as a condition that not only significantly impairs the physical and mental wellbeing of patients but also places a substantial financial burden on the healthcare system (Sharma and Rao, 2017).

In clinical practice, various laxatives are used to provide immediate relief from constipation symptoms, but they often lead to recurrence when discontinued. Furthermore, prolonged laxative use can result in drug dependence and worsen constipation symptoms, a phenomenon known as “laxative colon.” It is estimated that more than $800 million is expended annually on laxatives in the United States, with 20–30% of individuals aged 60 and older using laxatives on a weekly basis (Tanner et al., 2021). Therefore, the development of effective preventive and therapeutic strategies for STC is crucial for improving patient life quality and reducing healthcare costs.

Recent studies have shown that the pathogenesis of STC may be associated with anomalies in the enteric nervous system, Cajal interstitial cells, 5-hydroxytryptamine signaling, and the structural or functional aspects of colonic smooth muscle (Tillou and Poylin, 2017). Emerging evidence also implicates that an imbalance in gut flora plays a crucial role in the development of STC (He et al., 2020). Gut microflora, which are integral for maintaining gastrointestinal tract homeostasis, are involved in various physiological functions, such as food digestion, vitamin synthesis, bile acid transport, defense against pathogens, modulation of gastrointestinal immune responses, and regulation of epithelial cell metabolism (Sommer et al., 2017). Alterations in the relative abundance of beneficial and pathogenic bacteria in the gut flora can disrupt intestinal homeostasis, influencing the development and progression of STC (Wang and Yao, 2021). In 1998, a study discovered significant variations in gut flora between healthy individuals and those with constipation (Zoppi et al., 1998). Another study showed a reduction in the relative abundance of *Bifidobacteria* and *Lactobacillus* in constipated patients, accompanied by an increase in the relative abundance of the *Desulfovibrio* family (Zhuang et al., 2019). Furthermore, patients with constipation exhibited a significant decrease in short-chain fatty acid-producing bacteria, including *Faecalibacterium* and *Ruminococcaceae*, which corroborates the reduced levels of short-chain fatty acids, particularly butyric acid (He et al., 2020).

Metabolites produced by intestinal flora may play a key role in the interaction between intestinal flora and STC. Among these metabolites, short-chain fatty acids (SCFAs), primarily butyric acid, are important indicators for studying the relationship between intestinal microecology and constipation (Zhang et al., 2021). SCFAs, primarily butyric acid, have been shown to stimulate the synthesis of 5-hydroxytryptamine (5-HT) in enterochromaffin cells (ECs), thereby modulating intestinal motility. Certain intestinal flora have the ability to metabolize tryptophan into 5-HT, which in turn activates cAMP-dependent chloride channels in the colonic epithelium, thereby promoting intestinal secretion and transit (Bhattarai et al., 2018). These findings suggest that modulating intestinal flora and its metabolite SCFAs may be a new potential therapeutic approach for STC.

The Liqi Tongbian Decoction (LTD) is a traditional Chinese medicine formula comprising 10 herbs, including *Magnolia officinalis Rehd. et Wils*, *Aurantii Fructus Immaturus*, *Huomaren Cannabis sativa L.*, *Semen Pruni*, *Trichosanthes kirilowii Maxim*, *Raphani Semen*, *Radix Bupleuri*, *Paeoniae Radix Alba*, *Citrus reticulata*, and *Natrii sulfas* (Supplementary Table S1). Clinical studies have demonstrated that LTD effectively alleviates constipation symptoms, increases spontaneous bowel movements, and improves colonic transit in STC patients, particularly those with gas-stagnation syndrome (Liu et al., 2021). Furthermore, our previous study indicated that LTD modulates brain-gut peptide secretion in the treatment of functional constipation in rats with the Qi Stagnation Pattern (Tang et al., 2021). However, the effects of LTD on intestinal flora and metabolites in Qi stagnation STCs remain unexplored.

In this study, we induced the Qi Stagnation Pattern STC model using “loperamide hydrochloride + tail pinch stimulation” and investigated the therapeutic effects and underlying mechanism of LTD through the analysis of intestinal microorganisms and quantification of intestinal metabolites.

## Materials and methods

### Chemicals and reagents

Loperamide (Item No. H10910085) was obtained from Xian Janssen Pharmaceutical Co., Ltd. (Xi’an, China). Prucalopride succinate tablets (PST, Item No. H20183482) were purchased from Jiangsu Hansoh Pharmaceutical Group Co., Ltd. (Jiangsu, China). 5-HT ELISA kit was purchased from Jiangsu Enzyme Immunity Industry Co., Ltd. (Jiangsu, China). Antibodies specific to 5-HT (Item No. BS-1126R) and 5-HT4R (Item No. BS-2127R) were provided by Beijing Bo Aosen Biotechnology Co., Ltd. (BIOSS, Beijing, China). Primary antibodies specific to TPH1 (Item No. 41508) and TPH2 (Item No. 43424) were provided by Signalway Antibody LLC Co., Ltd. (SAB, USA). GAPDH (Item No. ABL1021) was used as an internal reference that was obtained from Abbkine Scientific Co., Ltd. (Wuhan, China).

### Preparation of Liqi Tongbian decoction

The medicinal liquid of LTD was obtained from the Chinese Medicine Preparation Room of the Second People’s Hospital affiliated with Fujian University of Traditional Chinese Medicine, and the chemical composition of this medicinal liquid was confirmed by ultra-high-performance liquid chromatography–tandem mass spectrometry (UHPLC-QE-MS), and the results of UHPLC-QE-MS are shown in Supplementary Table S2. Among the chemical constituents of LTD, flavonoids are the most abundant.

### Experimental animals and grouping

Thirty-six Wistar female rats, aged 6 weeks and weighing between 150 and 170 g, were purchased from the Beijing Huafukang Biotechnology Co., Ltd., Animal License: SCXK (Beijing) 2019-0008. The experimental rats were housed in cages in the Laboratory Animal Center of the Fujian University of Traditional Chinese Medicine. The rats were adaptively fed for 7 days in an environment with relative humidity of 40–60%, a 12-h light/dark cycle, and temperature of 22 ± 3°C. All animal experiments were performed in strict accordance with the guidelines of the Animal Management and Use Committee of Fujian University of Traditional Chinese Medicine (FJTCM IACUC 2022075).

After 1 week of adaptive feeding with a standard diet, the rats were randomly divided into six groups, each containing six rats. The groups were designated as follows: Ctrl (control) group, STC (model) group, LTD-LD group (low-dosage, 5.15 g/kg LTD), LTD-MD group (median-dosage, 10.3 g/kg LTD), LTD-HD group (high-dosage, 20.6 g/kg LTD), and positive control group (prukapril succinate tablet, PST).

### STC with Qi Stagnation Pattern model establishment and treatments

The STC rat model was induced by administering loperamide intraperitoneally at a dose of 3 mg/kg body weight twice daily for 14 days in all groups except the Ctrl group. Simultaneously, the tail-clamping stimulation method was employed to establish the Qi Stagnation Pattern model, inducing irritability and agitation in the rats. This stimulation was applied once daily for 30 min over a period of 14 days. The rats were observed for signs of agitation, irritability, dry and hard stools, and reduced defecation frequency during normal feeding. These signs indicated the successful modeling of constipation with the Qi Stagnation Pattern (Li et al., 2020). On the 15th day, the LTD-LD, LTD-MD, and LTD-HD groups received oral gavage of LTD at different dosages (5.15 g/kg, 10.3 g/kg, and 20.6 g/kg, respectively) 1 h after each loperamide administration for 14 days. The PST group was given prucalopride succinate tablets at a dose of 0.18 mg/kg·day by oral gavage 1 h after each loperamide administration for 14 days. The normal control group and model group were administered the same volume of sterile water *via* gavage at 1 mL/100 g body weight. One day before the end of the treatment period, rat fecal samples were collected, placed in sterile freezing tubes, and stored at −80°C for preservation for the detection of intestinal flora and SCFAs. Isoflurane inhalation anesthesia was administered, and samples of rat abdominal aortic artery blood and colon tissue were collected for subsequent experiments.

### Determination of water content in feces

At the end of the final treatment, rats were individually placed in metal cages, and fresh feces were collected every hour for 6 h. The feces were immediately weighed and then dried at 60°C for 6 h. The water content of the feces was calculated using the formula: (wet weight − dry weight)/wet weight × 100%.

### Measurement of intestinal transit rate

Colonic motility in rats was evaluated by tracking the movement of carbon ink through the intestines, and the small intestinal transit (%) was quantified. Each rat received 3 mL of intragastric gavage of 10% activated carbon 30 min after the final treatment. After 10 min, the rats were euthanized, and the small intestines between the pylorus and ileocecal region were collected and immediately extended to calculate the small intestinal transit. The intestinal transit rate was calculated as the percentage of the distance traveled by the activated carbon relative to the total length of the small intestine.

### Histopathological analysis of the colon

Colon tissues were fixed in 4% paraformaldehyde for 48 h, followed by dehydration, embedded in paraffin, and cut into slices of 4 μm thickness. Following deparaffinization, the sections were stained with hematoxylin and eosin (H&E) to observe the morphology of colon tissues. Additionally, Alcian Blue-Periodic Acid Schiff’s staining (AB-PAS staining) was used to detect the number of colonic goblet cells.

### Immunohistochemical staining analysis

Colon tissue slides, 4-μm-thick, were subjected to heat-induced antigen retrieval after dewaxing and hydration. Subsequently, the slides were incubated with primary antibody against 5-HT (1:100) or 5-HT4R (1:100) overnight at 4°C, followed by incubation with HRP-labeled secondary antibody and the addition of streptavidin–alkaline phosphatase solution for reaction. The reaction was developed with DAB and counterstained with hematoxylin. Five random fields on each slide were observed, and the optical density values were determined. Image analysis was conducted using Image-Pro Plus software (Media Cybernetics, Inc.).

### ELISA

At the end of the experiment, the blood from each rat was collected from the abdominal aorta. Serum 5-HT levels were determined using a Rat 5-HT ELISA kit following the manufacturer’s instructions. All the samples were analyzed in triplicate.

### Western bolt analysis

Total proteins were extracted from colon tissues using RIPA lysis buffer (Beyotime, Beijing, China), and protein concentrations were determined using the BCA protein assay kit (Vazyme, Nanjing, China). The samples were separated by 10% SDS-PAGE, transferred onto polyvinylidene fluoride (PVDF) membranes, and blocked with 5% skim milk for 2 h at room temperature. Subsequently, the samples were incubated with primary antibodies overnight at 4°C. The samples were then incubated with secondary antibodies for 2 h at room temperature. The protein expression was detected using a chemiluminescence detection system, and the band intensity was quantified using ImageJ. GAPDH was used as an internal reference.

### 16S rDNA sequencing and analysis

DNA was extracted from fecal samples using the E.Z.N.A.^®^ Stool DNA Kit (D4015, Omega, Inc., USA) according to the manufacturer’s instructions. The V3-V4 region was then amplified using the universal primers (341F: 5′-CCTACGGGNGGCWGCAG-3; 805R: 5′-GACTACHVGGGTATCTAATCC-3′). The samples were submitted to Shanghai Biotree Biotech Co., Ltd. (Shanghai, China) for pooling and paired-end sequencing on an Illumina MiSeq sequencer (Illumina). Samples were sequenced on an Illumina NovaSeq platform according to the manufacturer’s recommendations. Paired-end reads were assigned to samples based on their unique barcodes and trimmed by removing the barcode and primer sequence. Paired-end reads were merged using FLASH. Quality filtering was applied to the raw reads under specific criteria to obtain high-quality clean tags according to the fqtrim (v0.94) protocol. Chimeric sequences were removed using Vsearch software (v2.3.4). After dereplication with DADA 2, we obtained the feature table and feature sequence. Alpha diversity and beta diversity were calculated by normalizing the same sequences randomly. Feature abundance was normalized based on the relative abundance of each sample using the SILVA (release 132) classifier. Alpha diversity was assessed to analyze the complexity of species diversity within each sample, utilizing five indices: Chao1, observed species, goods coverage, Shannon, and Simpson. These indices were calculated using QIIME2. Beta diversity was calculated by QIIME2, and the graphs were generated using the R package. Blast was used for sequence alignment, and the feature sequences were annotated with the SILVA database for each representative sequence. Other diagrams were generated using the R package (v3.5.2).

### Fecal SCFA analysis

Fecal samples were dissolved in an EP tube containing 500 μL of methanol solution, allowed to stand for 5–10 min, and then shaken and mixed to make a fecal suspension. The pH of the suspension was adjusted to 2–3 with sulfuric acid and allowed to stand for 5 min with intermittent shaking and mixing. After the EP tubes were centrifuged at 5,000 r/min for 20 min, the supernatants were collected and centrifuged at 5,000 r/min for 5 min. And then the collected supernatants were used for gas chromatography–mass spectrometry (GC–MS) analysis. The column temperature was initially set at 100°C for 0.5 min, then increased to 180°C at a rate of 8°C/min for 1 min, and then increased to 200°C at a rate of 20°C/min for 5 min. The detector temperature was maintained at 240°C, the inlet temperature was set at 200°C, and the injection volume was 1 μL. The total detection time for each sample was 17.5 min.

### Statistical analysis

Data analysis was conducted using GraphPad Prism 8.0.1 (GraphPad Software Inc., United States) and SPSS 21.0. The data were expressed as mean ± SD. Differences between groups were evaluated using an analysis of variance. Correlations were assessed using the Pearson method. Statistical significance was defined as *p* < 0.05.

## Results

### Effects of LTD on general behavioral changes in STC rats with Qi Stagnation Pattern

**Figure 1:**
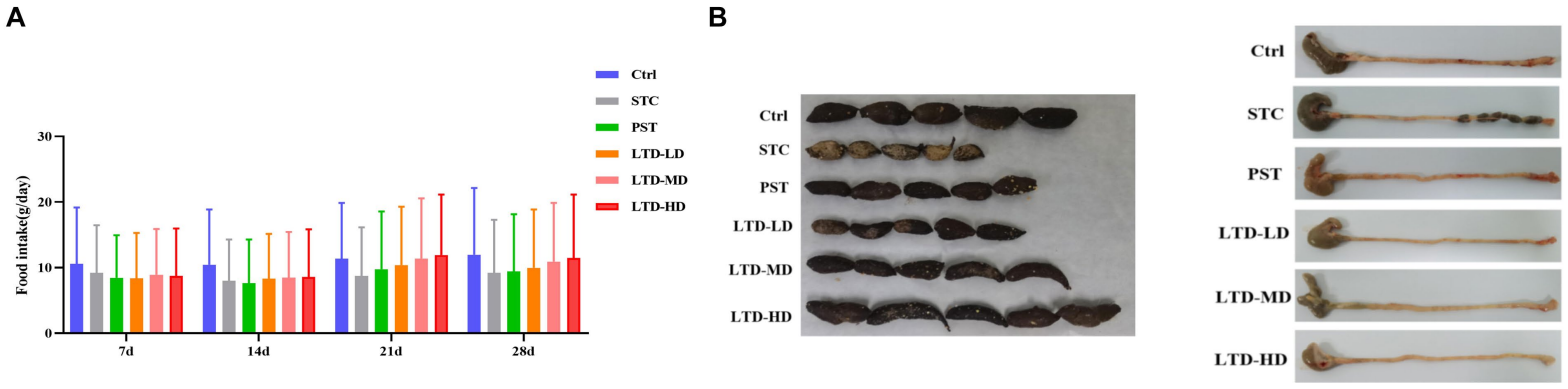
Effects of LTD on general behavioral changes in STC rats with Qi Stagnation Pattern. **(A)** Food intake, **(B)** comparison of fecal traits and residual colon feces in different groups of rats.

The rats in the STC group displayed irritability, reduced food intake, dull and discolored fur, slow weight gain, and produced small, hard feces. Following LTD treatment, improvements were observed in their general condition, food intake, mental state, and fecal characteristics. As shown in Figure 1A, STC rats with LTD treatment have a higher food intake and are more docile. Meanwhile, compared to the intestinal tract of STC rats with the Qi Stagnation Pattern, the treatment group contained fewer small, hard feces, and the cecum contents were substantial (Figure 1B).

### LTD improves constipation in STC rats with Qi Stagnation Pattern

**Figure 2:**
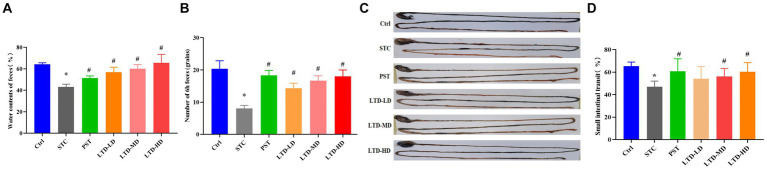
Improved defecation and intestinal transit in loperamide-induced STC rats by LTD. **(A)** Fecal water content, **(B)** number of feces in 6 h, **(C)** intestinal propelling movement of carbon ink, and **(D)** intestinal propulsive rate. *p < 0.05 vs. Normal, ^#^*p* < 0.05 vs. STC.

In comparison to the Ctrl group, the STC group exhibited a significant decrease in fecal water content and total fecal amount at 6 h (*p* < 0.05). LTD intervention led to an increase in both parameters compared to the STC group (*p* < 0.05). The PST group also showed improvements in these two parameters compared to the STC group (*p* < 0.05; Figures 2A,B). Medium and high doses of LTD significantly increased fecal water content compared to the PST group (*p* < 0.05). The colonic transit rate in the STC group was significantly slower compared with the Ctrl group (*p* < 0.05). However, it was significantly accelerated by medium and high doses of LTD (*p* < 0.05). In addition, the small intestinal transit rate in rats in the PST group was also significantly accelerated compared to that in the STC group (*p* < 0.05) (Figures 2C,D).

### Effect of LTD on the histopathology and goblet cells of colon in STC rats with Qi Stagnation Pattern

**Figure 3:**
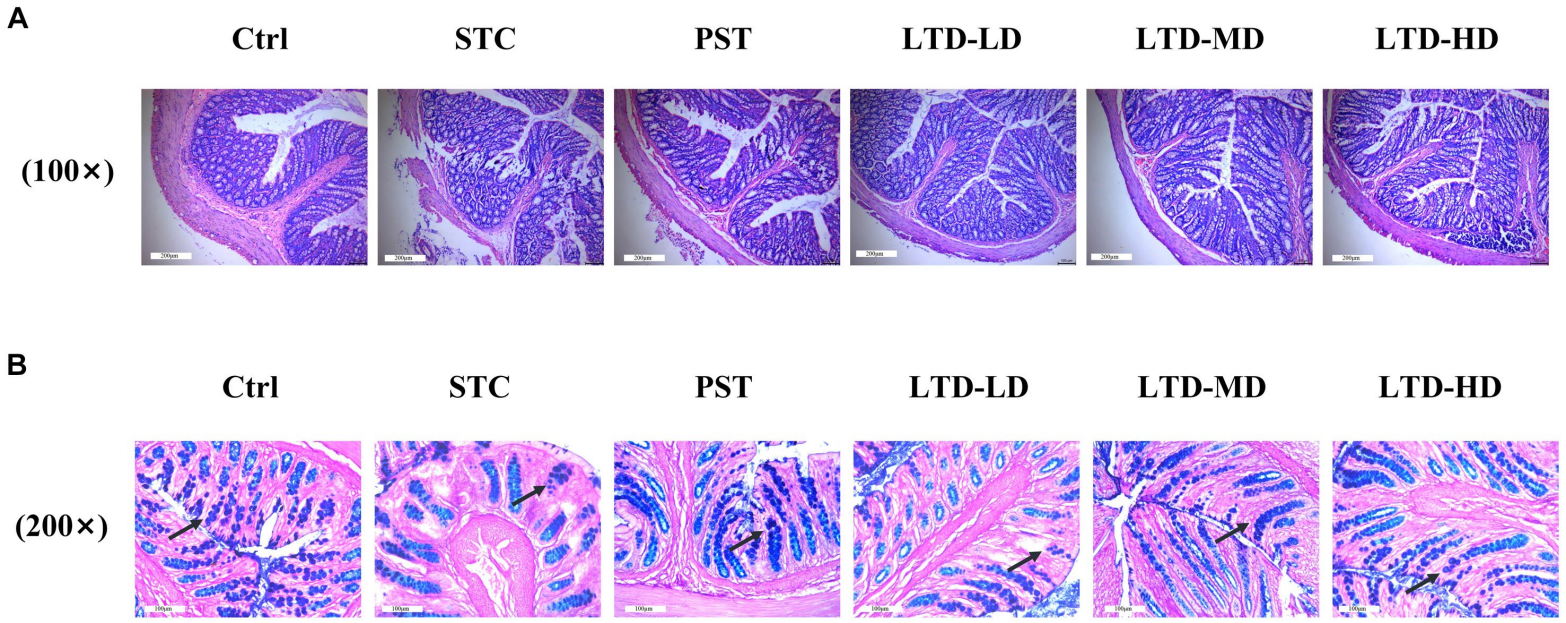
LTD improved the histopathology and goblet cell count of the colon in STC rats with the Qi Stagnation Pattern. **(A)** H&E staining results of colon tissue, **(B)** AB-PAS staining results of colon tissue.

H&E staining results revealed intact colonic mucosa and well-structured layers of the intestinal walls in the Ctrl group, with no inflammatory cell aggregation. In contrast, the STC group exhibited poorly aligned intestinal glands, detached intestinal epithelium, and dilated mesenchymal blood vessels in the submucosal layer of the colonic mucosa. Conversely, the LTD and PST groups displayed neatly aligned intestinal glands with no noticeable epithelial damage (Figure 3A). AB-PAS staining indicated atrophy and a reduced number of goblet cells in the STC group, whereas restoration was observed in the LTD and PST groups (Figure 3B).

### Effects of LTD on 5-HT and 5-HT4 receptor in STC rats with Qi Stagnation Pattern

**Figure 4:**
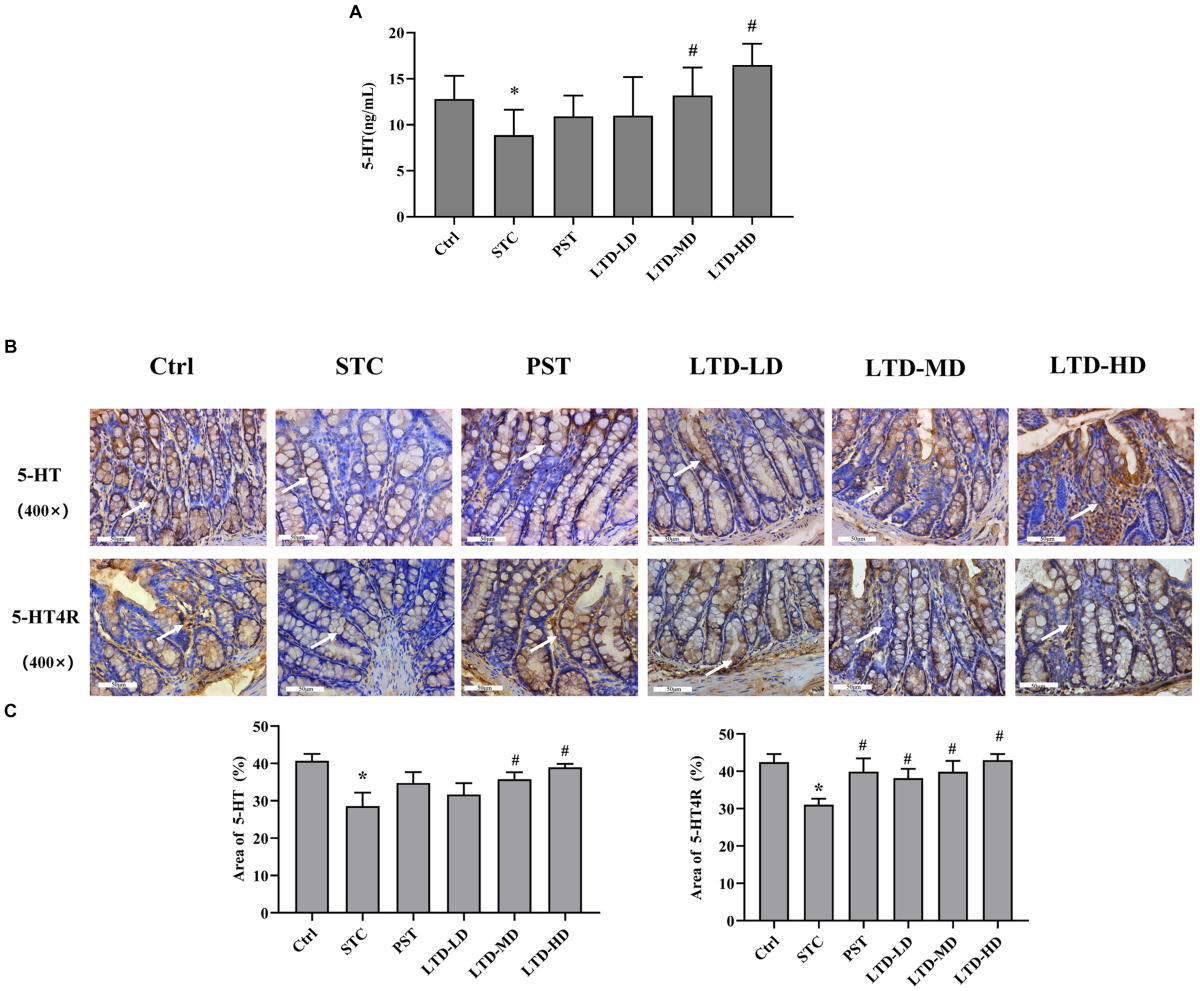
LTD promoted the secretion of 5-HT and activated its receptor pathway in the STC rats with the Qi Stagnation Pattern. **(A)** ELISA results of 5-HT in serum. **(B,C)** Immunohistochemical results of 5-HT and 5-HT4R in colon tissue. *p < 0.05 vs. Normal, ^#^*p* < 0.05 vs. STC.

The serum 5-HT concentration in the STC group was significantly lower than that in the Ctrl group (*p* < 0.05). Medium and high doses of LTD significantly elevated 5-HT content compared to the STC group (*p* < 0.05) (Figure 4A). The expression of 5-HT in the colonic mucosa of rats was significantly decreased in the STC group but significantly increased following the intervention of medium and high doses of LTD. The expression of 5-HT4R in the colonic mucosa of rats was significantly decreased in the STC group and significantly increased following the intervention of LTD and PST (Figures 4B,C).

### Effect of LTD on TPH1 and TPH2 protein levels in STC rats with Qi Stagnation Pattern

**Figure 5:**
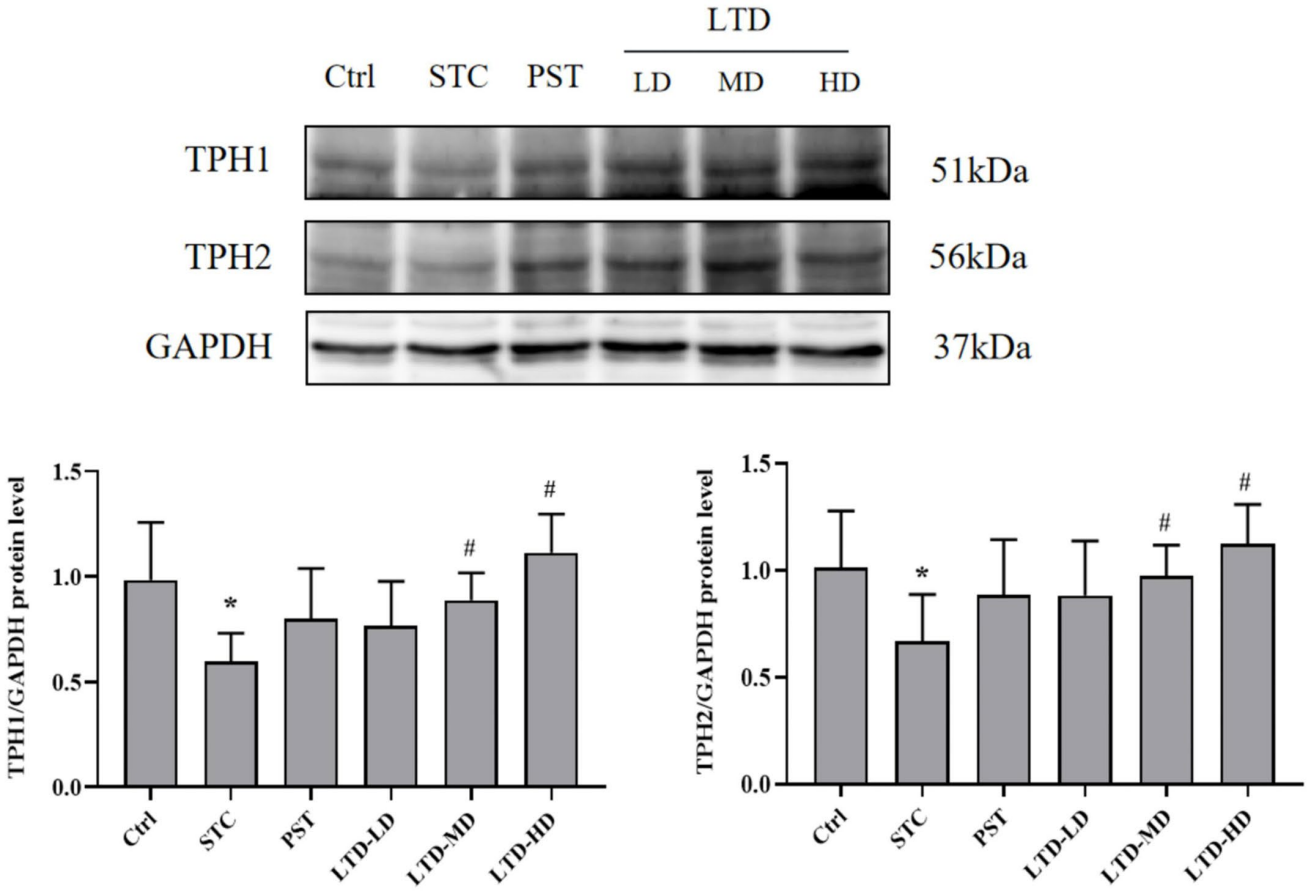
LTD upregulated TPH1 and TPH2 protein levels in STC rats with the Qi Stagnation Pattern. GAPDH was used as the internal standard. *p < 0.05 vs. Normal, ^#^*p* < 0.05 vs. STC.

Considering that LTD intervention significantly upregulated the 5-HT concentration in serum and protein expression in the colonic mucosa of STC rats with the Qi Stagnation Pattern, we further explored its effect on the protein expression of TPH1 and TPH2 during 5-HT synthesis. Western blot results indicated that the protein expression of TPH1 and TPH2 in colonic mucosa was significantly decreased in the STC group but significantly increased following the intervention of medium and high doses of LTD (*p* < 0.05) (Figure 5).

### Effects of LTD on colonic microflora in STC rats with Qi Stagnation Pattern

#### Effect of LTD on the diversity of gut microbiota

**Figure 6:**
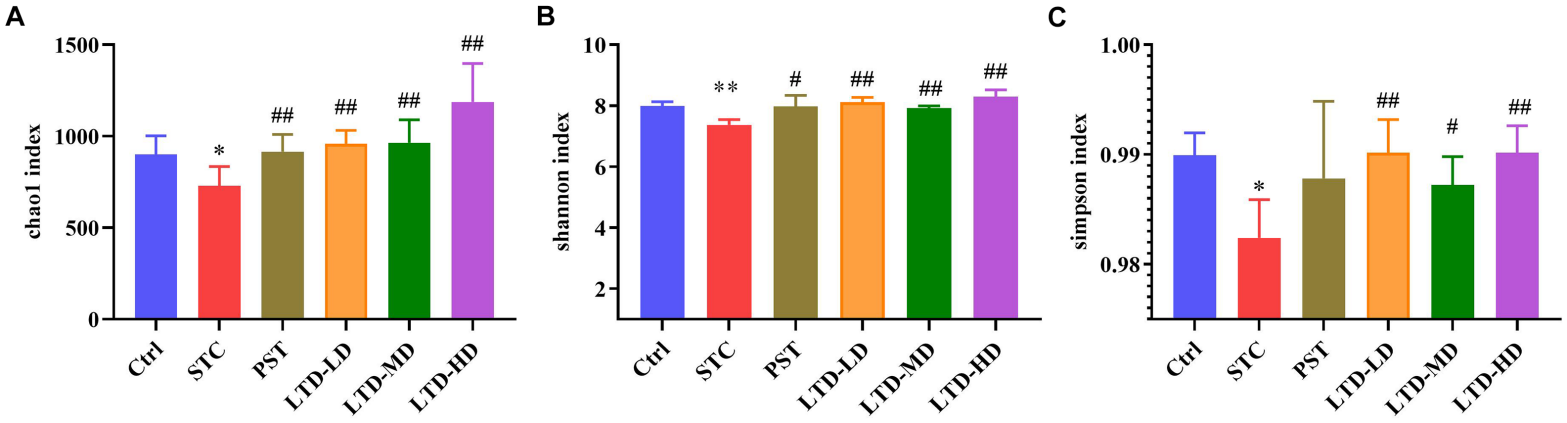
The alpha diversity of microbiomes identified in STC rats treated with LTD. The microbial abundances within the group were compared based on Chao1 **(A)**, Shannon **(B)**, and Simpson **(C)** indices separately. *p < 0.05, **p < 0.01 vs. Normal, ^#^*p* < 0.05, ^##^*p* < 0.01 vs. STC.

The diversity of the intestinal flora was assessed using the Chao1, Shannon, and Simpson indices. The Chao1 index was used to reflect the number of species contained in the community, i.e., the richness. Compared with the Ctrl group, the Chao1 index was decreased in the STC group (*p* < 0.05), indicating a significant reduction in species abundance in STC rats with Qi Stagnation Pattern. However, the Chao1 index was significantly elevated in the PST group and LTD group (*p* < 0.01) (Figure 6A). The Shannon and Simpson indices represent the diversity and uniformity of the intestinal flora. Both the Shannon and Simpson indices were significantly lower (*p* < 0.05) in the STC group compared with the Ctrl group, indicating reduced species diversity and homogeneity in STC rats with the Qi Stagnation Pattern. While the Shannon index increased in the PST group compared to the STC group (*p* < 0.05), no statistical difference was observed in the Simpson index (*p* > 0.05). Notably, the LTD group exhibited a significant increase in both the Shannon and Simpson indices compared to the STC group (*p* < 0.01). These results indicated that both STC rats with Qi Stagnation Pattern and after administration of LTD were able to affect the community structure of rat intestinal flora, and LTD was able to increase the diversity, abundance, and evenness of microbial populations (Figures 6B,C).

**Figure 7:**
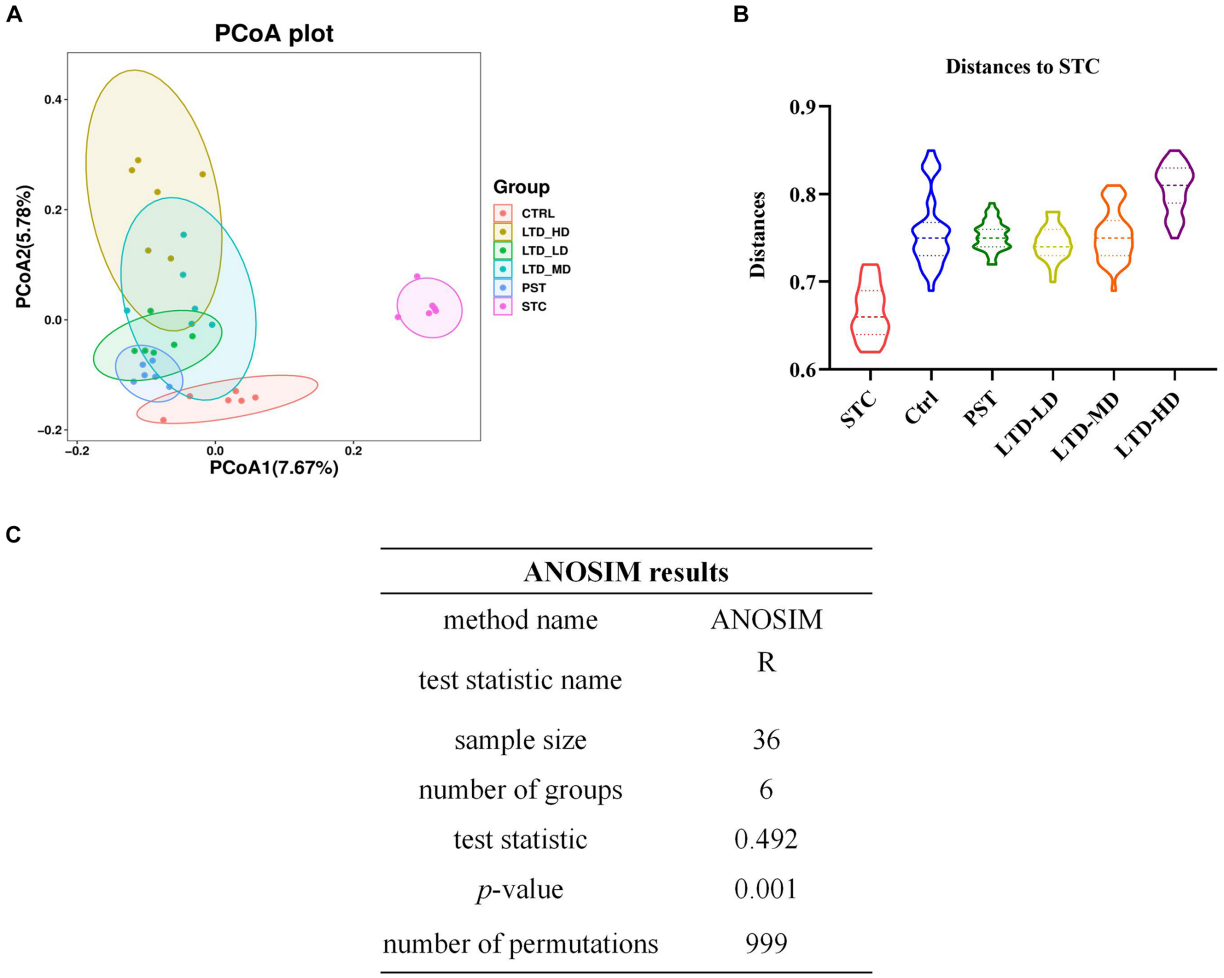
The beta diversity of microbiomes characterized in the STC model rats with LTD treatment. **(A)** PCoA analysis based on unweighted UniFrac, **(B)** distances to STC, and **(C)** ANOSIM method results.

Beta diversity analysis revealed that there was no overlap between the STC group and the Ctrl group, indicating significant differences in community structures between the normal rats and the STC rats (*p* < 0.01) (Figures 7A,B). The varying degrees of proximity and overlap among the LTD-LD, LTD-MD, and LTD-HD groups and the Ctrl group suggest that the intestinal flora compositions in each LTD dose group are converging toward normalcy. In addition, the magnitude of the distance between the different LTD dose groups and the STC group followed the order of LTD-HD > LTD-MD > LTD-LD, with each group showing a statistically significant difference in community structure compared to the STC group (*p* < 0.01). Further analysis using the ANOSIM method confirmed significant differences in microbial composition structure among the grouped samples (R-value: 0.492, *p*-value: 0.001) (Figure 7C). These results collectively suggested that the community structure of the STC group is significantly different from that of the other groups, with the divergence becoming more pronounced with higher doses of LTD.

#### Effect of LTD on the composition of intestinal flora

**Figure 8:**
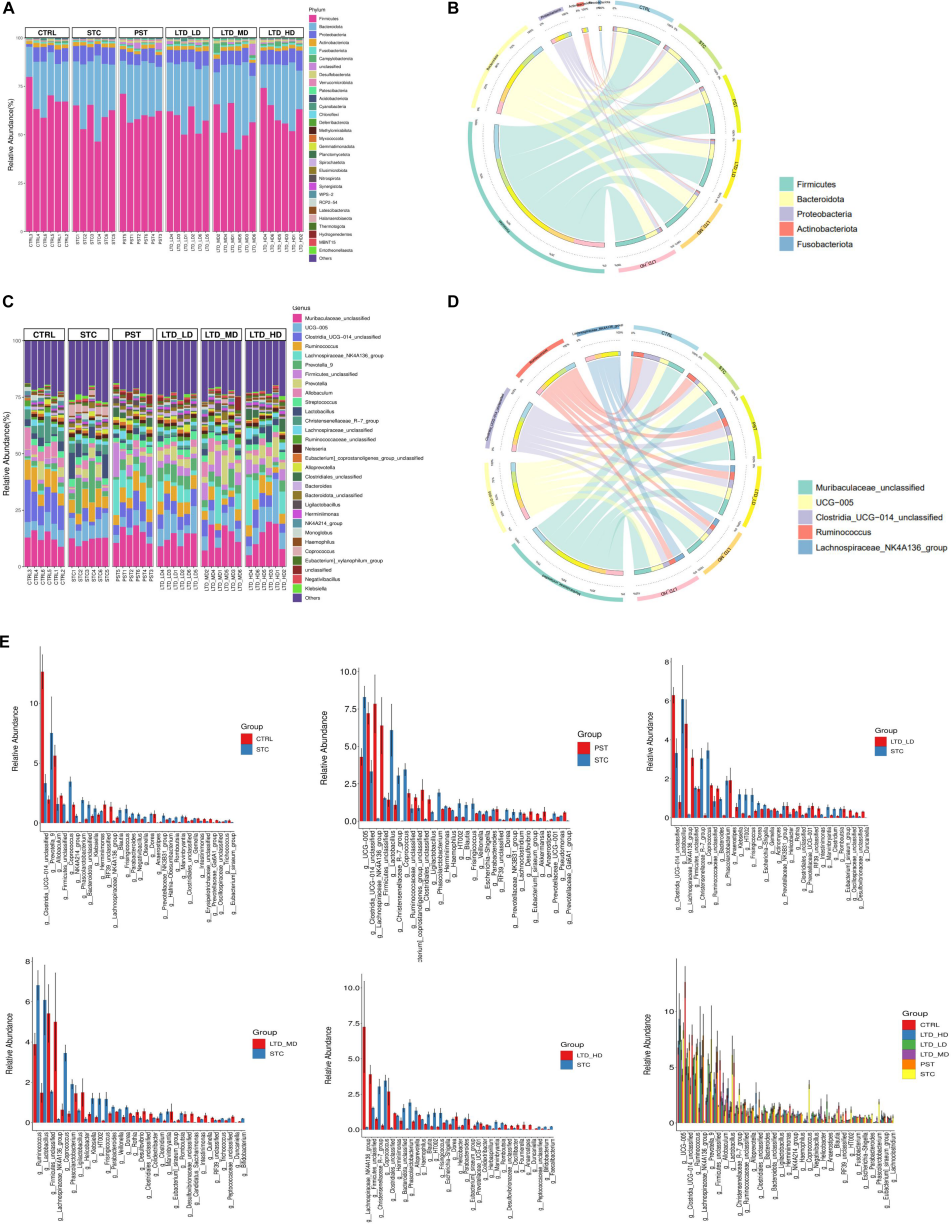
Microbiome alterations at various taxonomic levels in STC rats after LTD treatment. The microbiome profile alterations among the six mouse groups were compared at the levels of phylum **(A,B)** and genus **(C,D)**. **(E)** Comparison of intestinal flora between groups at the genus level.

To elucidate the compositional structure of the intestinal flora in each group at phylum and genus taxonomic levels, community bar charts were generated for visualization and analysis. At the phylum classification level (Figures 8A,B), the predominant phyla across all groups were Firmicutes, Bacteroidota, and Proteobacteria, collectively accounting for over 90% of the total abundance. Notably, STC rats with Qi Stagnation Pattern exhibited a decrease in Bacteroidota abundance (STC: 58.64% vs. Ctrl: 67.76%) and an increase in Bacteroidota (STC: 29.03% vs. Ctrl: 21.88%) and Proteobacteria (STC: 8.01% vs. Ctrl: 5.73%) phylum abundance compared to the Ctrl group. Following LTD administration, there was a progressive increase in Firmicutes (LTD-LD, LTD-MD, and LTD-HD: 55.27, 57.49, and 61.32%) abundance, with the high dose of LTD demonstrating the most pronounced impact.

At the genus classification level (Figures 8C,D), the top 10 genera identified in the intestinal flora of rats in each group, including Muribaculaceae, UCG-005, Clostridia, Ruminococcus, Lachnospiraceae_NK4A136, Prevotella_9, Firmicutes, Prevotella, Allobaculum, and Streptococcus. Notably, Lachnospiraceae_NK4A136 emerged as a key genus influenced by varying doses of LTD intervention. Comparative analysis (Figure 8E) revealed that in comparison to the Ctrl group, the relative abundance of 28 bacterial genera dominated by Prevotella_9, Coprococcus, and Phascolarctobacterium was increased in the STC group. Conversely, the relative abundance of 37 bacterial genera dominated by Clostridia, Allobaculum, and Firmicutes decreased in the STC group. Compared to the STC group, the low-dose LTD group exhibited an increase in the relative abundance of 55 genera dominated by Clostridia and Trichosporon spp. and a decrease in the relative abundance of 34 genera dominated by Christensenellaceae and Lactobacillus. The medium-dose LTD group showed an increase in the relative abundance of 39 bacterial genera, dominated by Firmicutes and Trichoderma spp., and a decrease in the relative abundance of 38 bacterial genera, dominated by Ruminococcus and Lactobacillus. In the high-dose LTD group, the relative abundance of 20 genera dominated by Trichosporon spp. and Firmicutes was increased, while 18 genera dominated by Christensenellaceae and Alloprevotella were decreased. Additionally, the PST group exhibited an increase in the relative abundance of 52 genera dominated by Lachnospiraceae and Clostridia, while 44 genera dominated by UCG-005 and Christensenellaceae_R-7 were decreased. These results highlighted that Lachnospiraceae_NK4A136 is the key genera influenced by different doses of LTD intervention.

**Figure 9:**
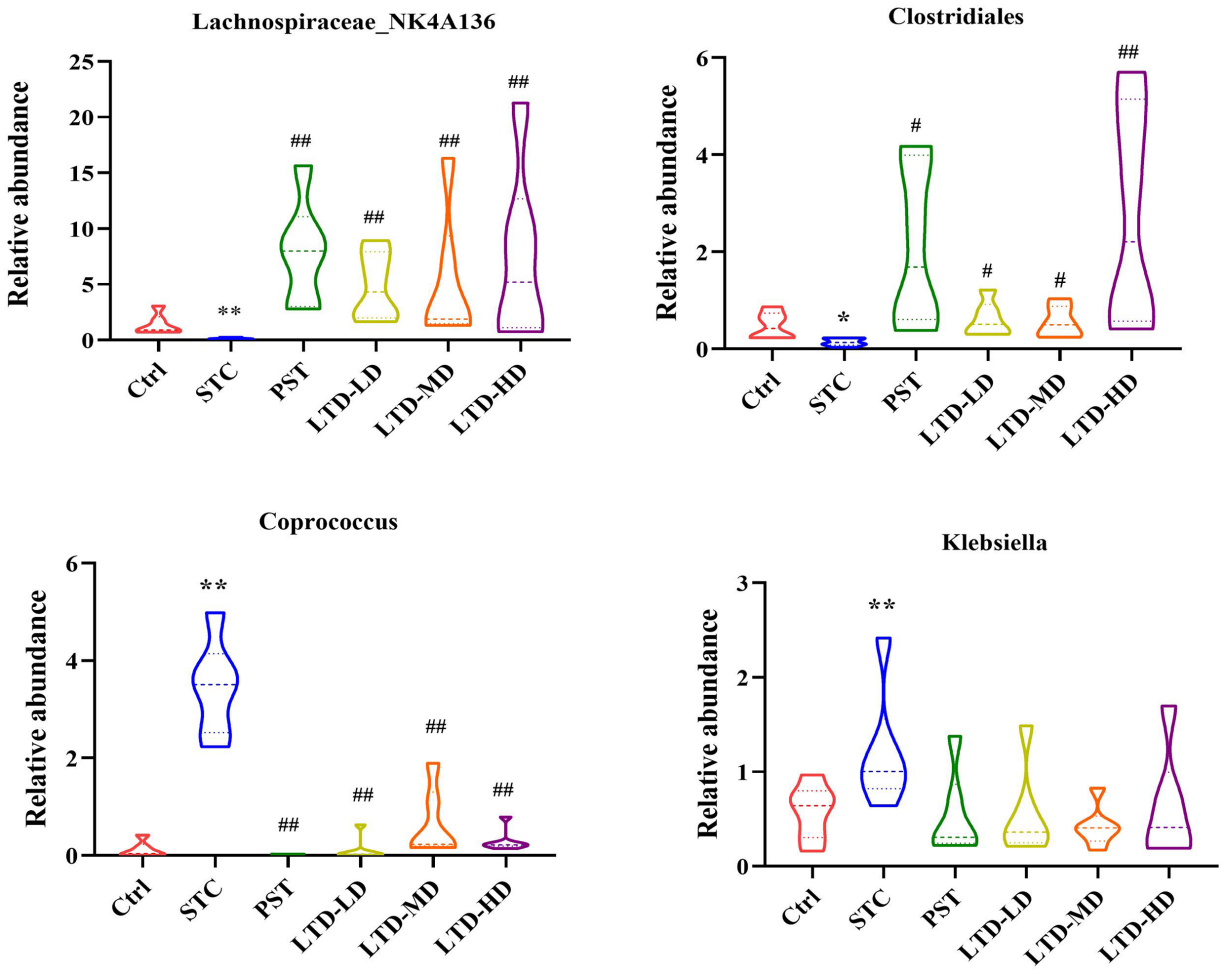
Relative abundances of Lachnospiraceae_NK4A136, Clostridiales, Coprococcus, and Klebsiella in fecal samples collected from STC rats treated with LTD. *p < 0.05, **p < 0.01 vs. Normal, ^#^*p* < 0.05, ^##^*p* < 0.01 vs. STC.

Significant genera were selected for further comparative analysis, and the results are shown in Figure 9. The abundance of Coprococcus and Klebsiella in the intestinal flora of the STC group was significantly higher than that in the Ctrl group. Both of them are considered conditional pathogens and have been associated with various intestinal dysfunctions, including intestinal dynamics, intestinal inflammation, and metabolic disturbances, among other diseases. Conversely, LTD intervention resulted in a decrease in the abundance of Coprococcus, with the high dose exhibiting the most substantial effect. Although there was also a decrease in the abundance of Klebsiella, this difference was not statistically significant. Moreover, the abundance of Lachnospiraceae_NK4A136 and Clostridiales in the intestinal flora of rats in the STC group was significantly lower than that in the Ctrl group. Both of them are beneficial bacteria. However, LTD intervention markedly increased the abundance of Lachnospiraceae_NK4A136 and Clostridiales, with the high dose demonstrating the most pronounced effect (*p* < 0.01).

In summary, the above results suggest that constipation has a substantial impact on the abundance of intestinal flora in rats. However, LTD intervention significantly increased the abundance of beneficial bacteria while decreasing the abundance of harmful bacteria. Notably, LTD intervention promoted the growth of short-chain fatty acid-producing bacteria, such as Lachnospiraceae_NK4A136 and Clostridiales, while reducing the abundance of bacteria linked to abnormal intestinal motility (e.g., Coprococcus), thereby contributing to the maintenance of intestinal flora homeostasis.

**Figure 10:**
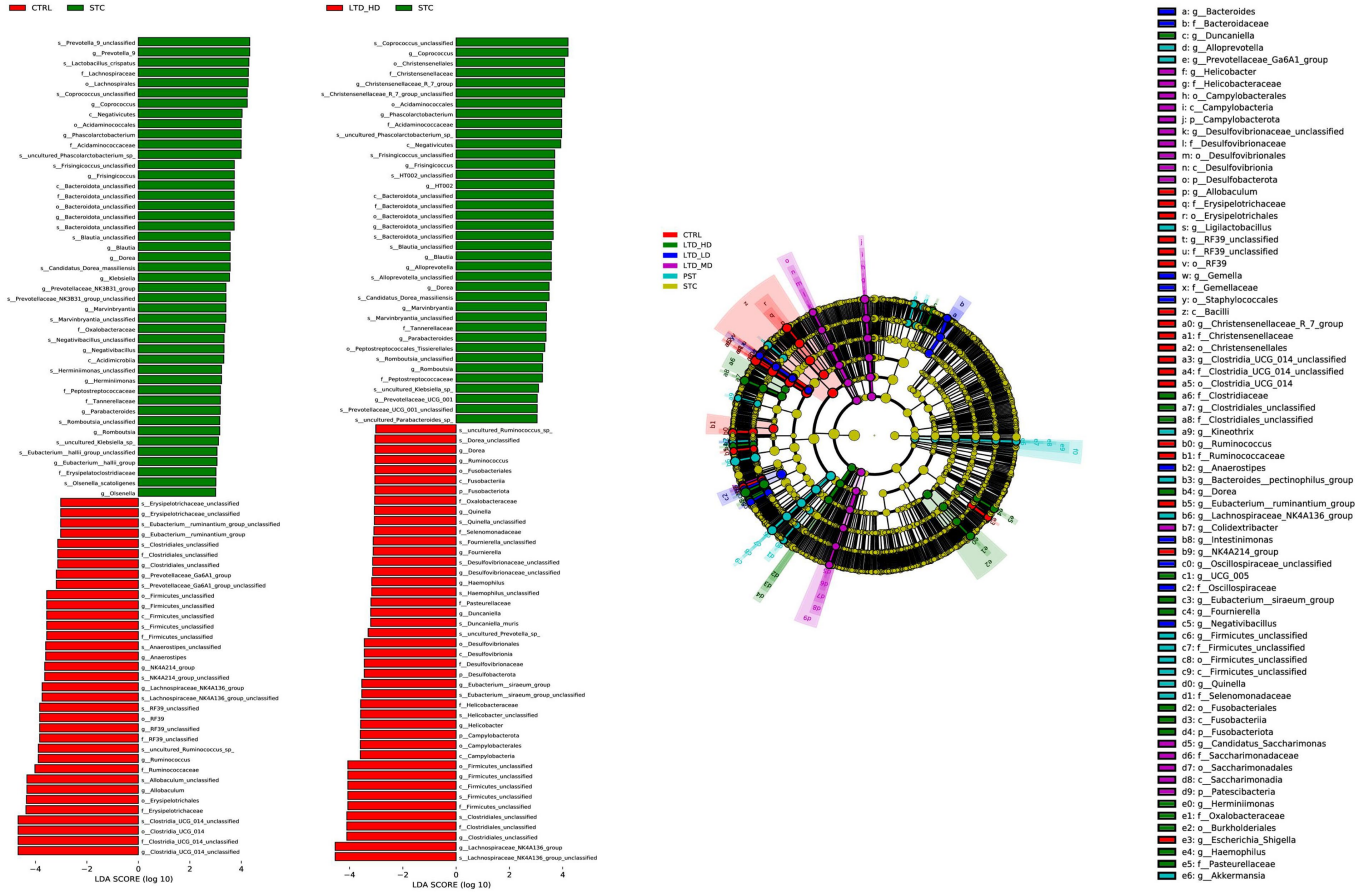
Taxonomic cladogram of major differential microbial species in different groups of rats through LEfSe analysis. Biomarker taxa are highlighted with colored circles and shaded areas. Each circle’s diameter reflects the abundance of those taxa in the community.

To further identify significantly distinct species, we employed linear discriminant analysis (LDA) and LEfSe analysis at the taxonomic level. The results are shown in Figure 10, highlighting that high-dose LTD significantly enriched SCFA-producing bacteria such as Trichoderma spp. and Clostridium spp. In contrast, the STC group exhibited enrichment of the conditionally pathogenic bacterium *E. faecalis* spp.

**Figure 11:**
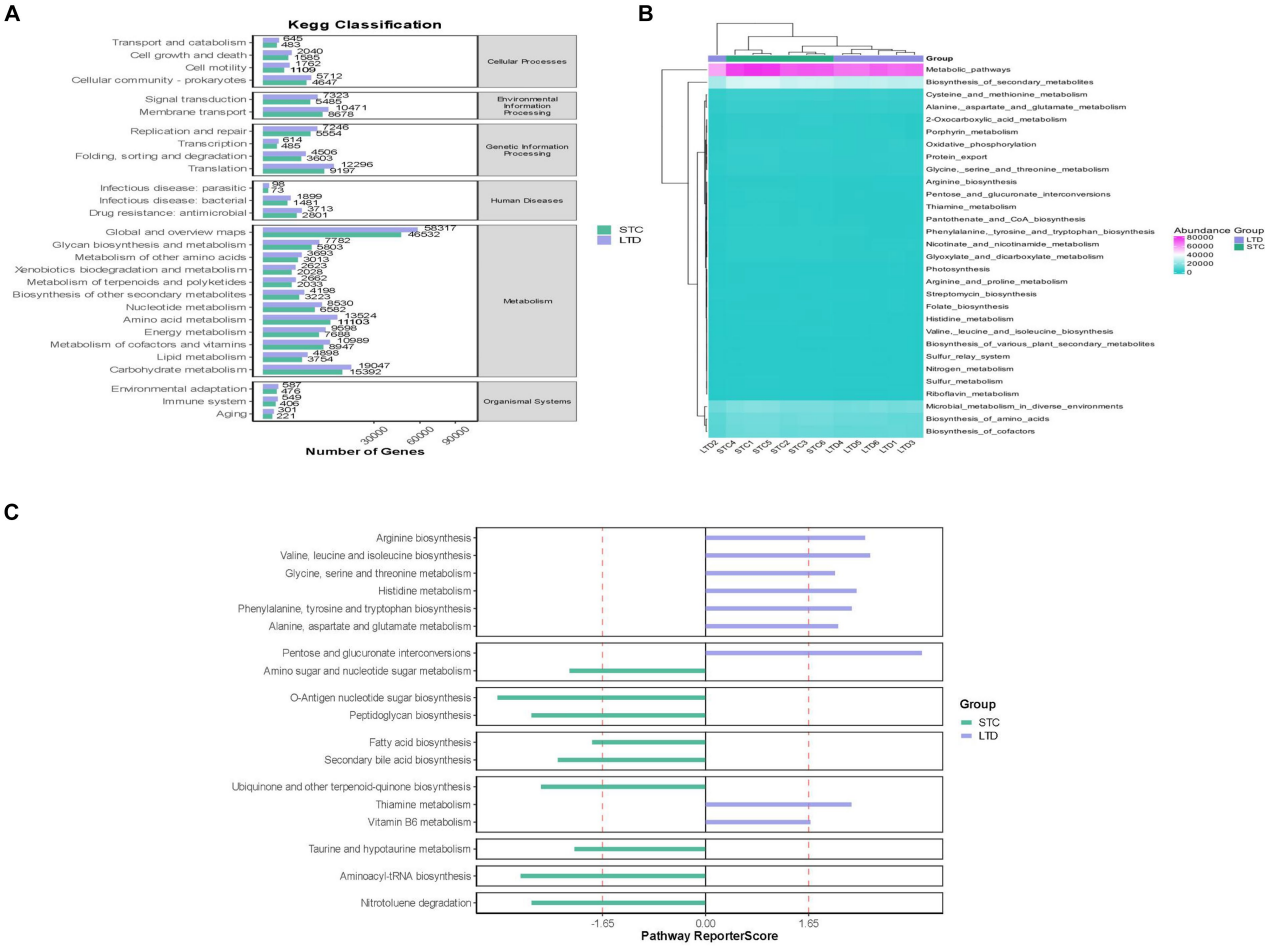
Functional prediction analysis (between the STC and LTD groups): **(A)** KEGG-level first function prediction. **(B)** KEGG-level secondary function prediction. **(C)** KEGG enrichment analysis.

Based on the above results, the STC group and the high-dose LTD group were selected for differential flora function prediction analysis, comparing the sequencing data with the KEGG database to elucidate corresponding pathways and functions. As shown in Figure 11, the KEGG level 1 function prediction encompassed six pathway functions, primarily associated with metabolism, cellular processes, and genetic information processing. In terms of secondary KEGG function prediction related to metabolism, the majority of genes in both groups were primarily annotated in the global and overview maps pathways, followed by pathways related to carbohydrate metabolism, amino acid metabolism, metabolism of cofactors, and vitamins. Importantly, the number of genes annotated in the LTD group was significantly greater than that in the STC group. Subsequently, KEGG enrichment analysis indicated a substantial upregulation in the activity of the pathways related to the biosynthesis of tryptophan, phenylalanine, and tyrosine, as well as histidine biosynthesis, following LTD intervention.

### Effects of LTD on SCFAs in colonic contents of STC rats with Qi Stagnation Pattern

**Figure 12:**
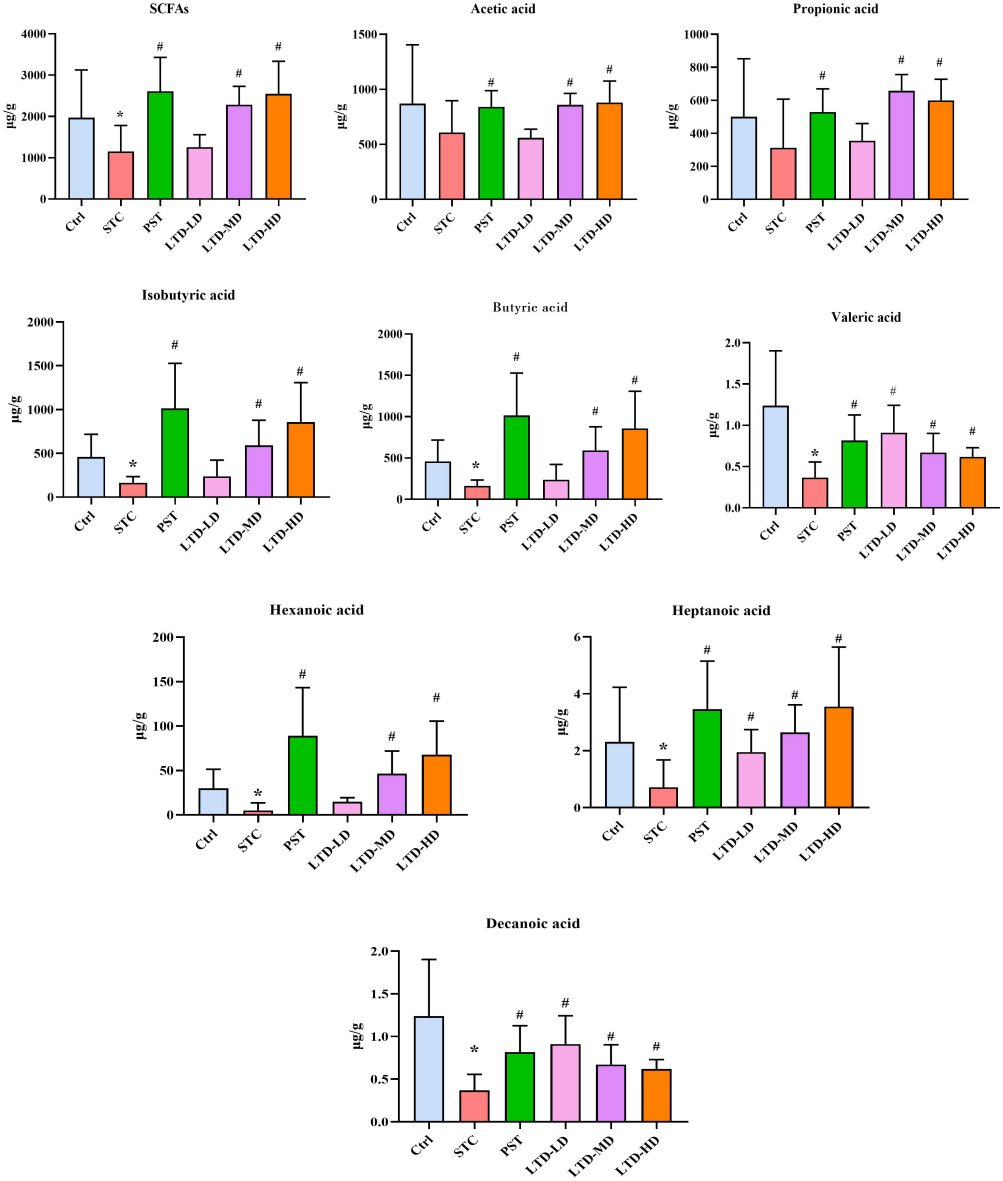
Effects of LTD on short-chain fatty acids (SCFAs) in the colonic contents of the STC rats with Qi Stagnation Pattern. *p < 0.05 vs. Normal, ^#^*p* < 0.05 vs. STC.

SCFAs are the end products of dietary fiber fermentation by intestinal flora, with acetic acid, propionic acid, and butyric acid being the most abundant, constituting over 90% of the total SCFAs. In the STC group, a significant reduction in the content of SCFAs was observed (*p* < 0.05); following the intervention, the PST, LTD-M, and LTD-H groups exhibited a notable elevation in SCFA content (all *p*s < 0.05). Specifically, the STC group exhibited a significant decrease in the contents of isobutyric acid, butyric acid, valeric acid, hexanoic acid, heptanoic acid, and decanoic acid (all *p*s < 0.05), while acetic acid and propionic acid showed a reduction that was not statistically significant (all *p*s > 0.05). Conversely, the PST group exhibited a significant increase in the levels of acetic acid, propionic acid, isobutyric acid, butyric acid, valeric acid, hexanoic acid, heptanoic acid, and decanoic acid (all *p*s < 0.05). Furthermore, the LTD-M and LTD-H groups showed significant increases in the contents of acetic acid, isobutyric acid, butyric acid, valeric acid, and hexanoic acid (all *p*s < 0.05), while heptanoic acid and decanoic acid in all the LTD groups, along with propionic acid in the LTD-M and LTD-H groups, displayed an increase (all *p*s < 0.05), but demonstrated a diminishing trend with escalating doses. SCFAs serve as crucial indicators for investigating the relationship between intestinal microecology and constipation, as they influence motility through stimulation of colonic mucosal receptors, the intestinal vagus nerve, or direct interaction with colonic smooth muscle. These findings confirmed that LTD substantially elevates SCFA levels, especially butyric acid (Figure 12).

### Spearman correlation analysis between the SCFAs, 5-HT, and the colonic microflora

#### Correlation analysis of differential genera with differential SCFAs

**Figure 13:**
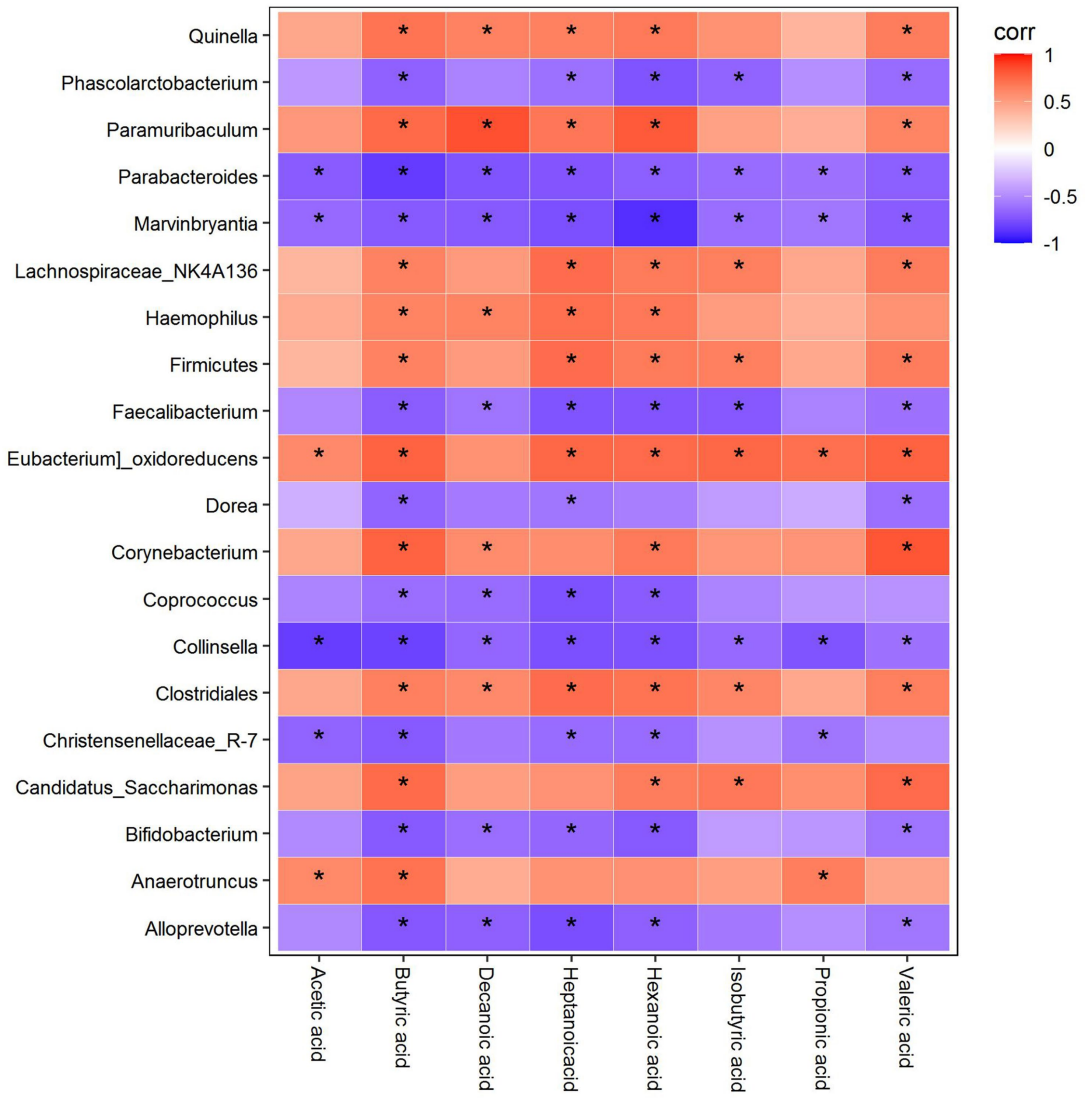
Spearman correlation analysis between the SCFAs, 5-HT, and the colonic microflora.

As shown in Figure 13, Quinella was positively correlated with butyric acid, decanoic acid, heptanoic acid, hexanoic acid, and valeric acid, while Phascolarctobacterium was negatively correlated with butyric acid, heptanoic acid, hexanoic acid, isobutyric acid, and valeric acid. Paramuribaculum was positively correlated with butyric acid, decanoic acid, heptanoic acid, hexanoic acid, and valeric acid, whereas Parabacteroides and Marvinbryantia were negatively correlated with acetic acid, butyric acid, decanoic acid, heptanoic acid, hexanoic acid, isobutyric acid, propionic acid, and valeric acid. Lachnospiraceae_NK4A136 was positively correlated with butyric acid, heptanoic acid, hexanoic acid, isobutyric acid, and valeric acid, while Haemophilus was positively correlated with butyric acid, decanoic acid, heptanoic acid, and hexanoic acid. Firmicutes were positively correlated with butyric acid, heptanoic acid, hexanoic acid, isobutyric acid, and valeric acid, while Faecalibacterium was negatively correlated with butyric acid, decanoic acid, heptanoic acid, hexanoic acid, isobutyric acid, and valeric acid. Eubacterium_oxidoreducens was positively correlated with acetic acid, butyric acid, heptanoic acid, hexanoic acid, isobutyric acid, propionic acid, and valeric acid, while Dorea was negatively correlated with butyric acid, heptanoic acid, and valeric acid. Corynebacterium was positively correlated with butyric acid, decanoic acid, hexanoic acid, and valeric acid, whereas Coprococcus was negatively correlated with butyric acid, decanoic acid, heptanoic acid, and hexanoic acid. Collinsella was negatively correlated with acetic acid, butyric acid, decanoic acid, heptanoic acid, hexanoic acid, isobutyric acid, propionic acid, and valeric acid, while Clostridiales was positively correlated with butyric acid, decanoic acid, heptanoic acid, hexanoic acid, isobutyric acid, and valeric acid. Christensenellaceae R-7 was negatively correlated with acetic acid, butyric acid, heptanoic acid, hexanoic acid, and propionic acid, while Candidatus_saccharimonas was positively correlated with butyric acid, acetic acid, isobutyric acid, and valeric acid. Bifidobacterium was negatively correlated with butyric acid, decanoic acid, heptanoic acid, hexanoic acid, and valeric acid. Anaerotruncus was positively correlated with acetic acid, butyric acid, and propionic acid, while Alloprevotella was negatively correlated with butyric acid, decanoic acid, heptanoic acid, hexanoic acid, and valeric acid (Figure 13).

#### Correlation analysis between differential genera, differential SCFAs, and 5-HT

To further clarify the relationship among intestinal flora, SCFA, and 5-HT concentration, Spearman correlation analysis was employed to calculate correlation coefficients and their *p*-values for different genera, SCFA, and 5-HT concentration. The results are shown in Supplementary Tables S3, S4: (1) The correlations between different bacterial genera and 5-HT concentrations were compared as follows: Lachnospiraceae_NK4A136, Firmicutes, Clostridiales, Haemophilus, Quinella, Eubacterium_oxidoreducens, Paramuribaculum, Corynebacterium, Anaerotruncus, and Candidatus_Saccharimonas were positively correlated with 5-HT concentrations. Conversely, Christensenellaceae_R-7, Alloprevotella, Dorea, Parabacteroides, Coprococcus, Bifidobacterium, Marvinbryantia, Phascolarctobacterium, Collinsella, and Faecalibacterium were negatively correlated with 5-HT concentrations. (2) Differential SCFA and 5-HT concentrations were compared as follows: isobutyric acid, butyric acid, valeric acid, hexanoic acid, heptanoic acid, and decanoic acid were positively correlated with 5-HT concentrations.

## Discussion

Slow transit constipation (STC) is a prevalent condition characterized by delayed colonic peristalsis and impaired intestinal content expulsion, which significantly impact patients’ physical and mental wellbeing, overall quality of life, and potentially lead to other gastrointestinal complications. In the present study, we investigated the mechanisms underlying the therapeutic effects of LTD in treating STC with Qi stagnation, focusing on its impact on 5-HT, the 5-HT4R receptor, intestinal flora, and short-chain fatty acids in STC rats. Our primary findings can be summarized below: (1) Improved Fecal Characteristics: LTD administration resulted in enhanced fecal characteristics, increased defecation frequency, elevated fecal water content, and accelerated intestinal propulsion in STC rats with Qi Stagnation Pattern; (2) Colon Pathology Amelioration: LTD treatment ameliorated colon pathology in STC rats, which including more organized intestinal glands, increased the number of cup cells, and enhanced intestinal mucus secretion; (3) Activation of 5-HT Signaling Pathway: LTD played a role in activating the 5-HT signaling pathway. It upregulated the protein expression of TPH1 and TPH2 in colonic tissues, which are facilitated 5-HT synthesis; (4) Enhanced Intestinal Flora Diversity and Abundance: LTD enhanced the diversity, abundance, and homogeneity of intestinal flora, highlighting Lachnospiraceae_NK4A136 spp. as the key genus that responded positively to LTD treatment. (5) Functional prediction: Functional prediction analysis revealed an emphasis on the metabolic pathway, particularly an increase in the activity of tryptophan synthesis pathway. This pathway is important for the production of serotonin (5-HT) and could explain the observed upregulation of 5-HT synthesis. (6) SCFA Metabolism: LTD treatment had a notable impact on SCFA metabolism in STC rats with Qi Stagnation Pattern. It increased the total SCFAs and upregulated the contents of various SCFAs, with a notable increase in butyric acid; (7) Correlations: Positive correlations were observed between specific genera, such as Trichoderma spp. and Clostridium spp., with both butyric acid and 5-HT concentrations. Additionally, enhanced butyric acid production was correlated with increased levels of Lachnospiraceae_NK4A136 and Clostridiales.

The establishment of a simple, stable, and reliable animal model that mimics the clinical manifestations of STC is crucial for a comprehensive understanding of its pathogenesis (Sharma and Rao, 2017), underlying mechanisms (Yao et al., 2022), and potential therapeutic interventions (Vlismas et al., 2024). It’s also essential for understanding the pharmacodynamic mechanisms of LTD in the treatment of STC. Loperamide, an opioid receptor agonist, is widely recognized for use in STC modeling. It inhibits intestinal smooth muscle contraction, reduces acetylcholine release, and directly inhibits intestinal peristalsis. Additionally, it elevates the expression of aquaporin 8, which facilitates transmembrane water transport, ultimately leading to reduced fecal water content. This model is mainly characterized by the presence of small fecal pellets, diminished fecal water content, decreased fecal SCFAs, and an imbalance of the intestinal flora (Wu et al., 2014). In the context of Chinese medicine, the liver is attributed to the functions of detoxification and excretion, and its fifth element is wood, which tends to be organized but dislikes being depressed. The cause of the disease is attributed to “depression and anger,” which disrupt the detoxification and excretion functions of the liver, leading to Qi stagnation. The “pinch the tail to provoke anger method,” inducing chronic stress, is a prevalent technique in China to create animal models exhibiting Qi stagnation (Jin et al., 2003; Zhou Guo'er et al., 2014).

In this study, we established a rat model of STC with the Qi Stagnation Pattern using a combination of loperamide administration and tail-clamping provocation. This approach allows us to investigate how LTD ameliorates STC with the Qi Stagnation Pattern. Our findings demonstrated that LTD increased the number, weight, and water content of fecal pellets, improved the rate of intestinal propulsion, and provided significant relief from constipation. Notably, the effects observed in the high-dose LTD group were almost indistinguishable from those in the positive control group.

Intestinal flora is a vast and complex micro-ecosystem within the organism that colonizes the human gut for a prolonged period while maintaining a symbiotic relationship with the host. A diverse array of microbiota substantially colonizes the adult gastrointestinal tract in substantial numbers. Numerous studies have demonstrated the disruption of intestinal flora in STC patients, underscoring its significant influence on STC pathogenesis. STC patients often exhibit a reduced abundance of beneficial flora and an increase in *Bacillus anisopliae* spp. (Dimidi et al., 2017). Thus, it is crucial to explore whether LTD treatment can ameliorate Qi Stagnation Pattern-associated STC by modulating intestinal flora. Our data indicated an increase in the richness, diversity, and homogeneity of the intestinal flora in rats following LTD intervention, indicating that the formula had a regulatory impact on the structure of the intestinal flora at multiple levels. In the analysis of bacterial abundance sequencing results, the primary focus was on the phylum and genus levels. Compared with the model group, the high-dose LTD (the most effective dose) increased the relative abundance of six phyla, primarily dominated by Campylobacterota and Fusobacteriota. Additionally, it resulted in a decrease in the relative abundance of one phylum, mainly dominated by the Elusimicrobiota, at the phylum level in the rat intestinal flora. At the genus level, compared with the model group, rats in each LTD group exhibited an increased abundance of Lachnospiraceae_NK4A136, Clostridiales, and Firmicutes, along with a decreased abundance of Coprococcus, Christensenellaceae_R-7, Dorea, and others. Notably, the Lefse analysis identified Lachnospiraceae_NK4A136 and Clostridiales as biomarkers for each dose group of LTD. The findings suggested that LTD can partially reverse the intestinal flora structure of STC rats and promote the growth/proliferation of potential probiotics.

Furthermore, to comprehend the function of the differential flora, we conducted functional prediction, revealing that the KEGG pathway was predominantly enriched in the metabolic pathway. Following LTD intervention, there was a significant increase in the pathways related to the biosynthesis of tryptophan, phenylalanine, tyrosine, arginine, and the metabolism of histidine. Studies have indicated that amino acids participate in protein digestion and absorption pathways, producing a considerable amount of SCFAs during the process. The intestinal flora produce SCFAs by fermenting undigested and absorbed carbohydrates, utilizing them as substrates to energize the organism’s cells. Acetic acid primarily supplies muscles, propionic acid benefits the liver, and butyric acid nourishes the colon epithelial cells. SCFAs exert a profound influence on the organism’s energy metabolism, mucosal growth, and cellular differentiation, significantly impacting how intestinal flora affects the host’s function and structure. Our study demonstrated that LTD-induced alterations in intestinal SCFAs in rats STC with Qi Stagnation Pattern, with butyric acid exhibiting the most notable changes. SCFAs, a prominent class of bacterial metabolites, directly activate G-protein-coupled receptors, inhibit histone deacetylases, and serve as energy substrates, thereby playing a crucial role in regulating host physiology, including gut motility. Butyric acid, in particular, contributes to maintaining the homeostasis of the colonic mucosa and modulating the excitability of enteric neurons. Previous research by Soret et al. revealed that butyric acid increased the population of choline acetyltransferase-positive neurons and stimulated the contraction of colonic smooth muscle, thereby promoting intestinal motility (Soret et al., 2010). Moreover, studies have reported that butyric acid can upregulate the expression of TPH1mRNA in enterochromaffin cells, leading to increased synthesis and release of 5-HT, consequently enhancing colonic motility (Canani et al., 2011). Transplantation of feces from STC patients into germ-free mice induced constipation symptoms and reduced the concentration of butyric acid (Ge et al., 2017). However, supplementation with butyric acid alleviated constipation-related symptoms in the mice. These findings suggest that butyric acid possesses potential therapeutic agent for constipation.

Additionally, we identified a positive correlation between Lachnospiraceae_NK4A136 spp. and Clostridium spp. with both butyric acid and 5-HT. The genus Lachnospiraceae_NK4A136 spp., classified within the Thick-walled Bacteria phylum, is commonly found in the intestinal tract of most healthy individuals. It is a potentially beneficial bacterium involved in the metabolism of a wide range of carbohydrates, especially pectin, a complex dietary fiber and prebiotic present in fruits and vegetables. Pectin is highly effective in producing butyric and acetic acid during fermentation, serving as significant energy sources for the host (Jiang et al., 2020). Furthermore, Lachnospiraceae_NK4A136 spp. predominantly producing butyric acid, is believed to induce intestinal mucosal growth, inhibit colonic mucosal inflammation, reduce the relative abundance of *Vibrio desulfuricans*, and modulate the body’s immune response (Zhu et al., 2019). Clostridiales, a group of intestinal flora capable of forming spores, belong to the anaerobic Bacillus spp. Yano et al. found that Clostridium spp. stimulated the secretion and release of 5-HT from enterochromaffin cells, leading to a significant increase in colonic and serum 5-HT levels, thereby enhancing gastrointestinal motility (Yano et al., 2015). In our study, LTD intervention increased the relative abundance of Trichoderma spp. and Clostridium spp. As a result, we postulated that Lachnospiraceae_NK4A136 and Clostridiales are the specific genera targeted by LTD to promote an increase in 5-HT content and enhance intestinal motility.

Tryptophan, an essential amino acid that cannot be synthesized within the body and must be obtained from dietary proteins, serves as the primary precursor for 5-HT synthesis and is hydrolyzed by the liver. Tryptophan hydroxylase (TPH) is an enzyme involved in tryptophan metabolism and is often used as an indirect indicator of 5-HT synthesis (Bertrand and Bertrand, 2010). TPH possesses two isoforms: TPH1, predominantly expressed in non-neuronal cells, and TPH2, found mainly in neuronal cells, including enteric neurons and central nervous system neurons. In physiological processes, tryptophan is catalyzed by TPH to form 5-hydroxytryptophan, which is further converted into 5-HT through levo-aromatic amino acid decarboxylase. Once synthesized, 5-HT is stored within cells, and released upon stimulation by physicochemical signals. It subsequently binds to receptors, eliciting corresponding responses (Cao et al., 2017). The 5-HT4R belongs to the G-protein-coupled receptors family and is expressed in both the submucosal and intermuscular plexus of the colon. Activation of 5-HT4R triggers the opening of voltage-sensitive calcium channels, facilitating the release of neurotransmitters such as substance P and acetylcholine. These neurotransmitters play crucial roles in gastrointestinal sensation and motility, ultimately inducing the contraction of colonic smooth muscle cells, thereby regulating colonic motility (Gwynne and Bornstein, 2019). In this study, we observed that LTD increased the protein expression of 5-HT4R, TPH1, and TPH2 in the colon, suggesting that LTD may activate the 5-HT pathway, enhance 5-HT synthesis, and promote intestinal motility in combination with 5-HT4R.

## Conclusion

The present study provides further evidence that LTD can ameliorate constipation in rats STC with Qi stagnation by modulating the structure of the intestinal flora and enhancing the secretion of SCFAs. LTD promotes the production of SCFAs, notably butyric acid, by increasing the abundance of Lachnospiraceae_NK4A136 and Clostridiales. Consequently, this process stimulates 5-HT synthesis, enhances colonic transport function, and contributes to the therapeutic efficacy of TLD in addressing Qi stagnation-related STC. This study provides novel insights into the therapeutic potential of LTD, considering the perspective of intestinal flora and its metabolite, SCFAs.

## Data availability statement

The datasets presented in this study can be found in online repositories. The names of the repository/repositories and accession number(s) can be found at: NCBI – PRJNA1074105.

## Ethics statement

The animal studies were approved by the Ethics Committee of Fujian University of Traditional Chinese Medicine. The studies were conducted in accordance with the local legislation and institutional requirements. Written informed consent was obtained from the owners for the participation of their animals in this study.

## Author contributions

QL: Writing – original draft. DK: Writing – original draft. YC: Writing – review & editing. AS: Methodology, Writing – review & editing. LL: Data curation, Writing – review & editing. LH: Writing – original draft, Formal analysis. YR: Data curation, Methodology, Writing – review & editing. WF: Formal analysis, Writing – review & editing. PZ: Data curation, Methodology, Writing – review & editing. TS: Writing – review & editing. YL: Funding acquisition, Writing – review & editing. XK: Funding acquisition, Writing – review & editing.
