# Supplementary material for: Effects of Liqi Tongbian decoction on gut microbiota, SCFAs production, and 5-HT pathway in STC rats with Qi Stagnation Pattern
Source: Front Microbiol. 2024 Mar 15;15:1337078. doi: 10.3389/fmicb.2024.1337078 (PMC10978654; doi:10.3389/fmicb.2024.1337078)
Supplement: Supplementary file 1 [file Table_1.DOCX]

Supplementary Material

| **Table 1** Detailed information of herbs in LTD. | | | | |
| --- | --- | --- | --- | --- |
| **Chinese name** | **Latin name** | **Plant names** | **Medicinal part** | **Amount (g)** |
| Houpu | *Magnolia Officinalis Rehd Et Wils．* | Magnolia officinalis Rehder & E.H.Wilson | Bark | 10 |
| Zhishi | *Aurantii Fructus Immaturus* | Citrus × aurantium L. | Fruit | 10 |
| Huomaren | *Huomaren Cannabis Sativa L.* | Cannabis sativa L. | Fruit | 15 |
| Yuliren | *Semen Pruni* | [Prunus humilis Bunge](https://mpns.science.kew.org/mpns-portal/plantDetail?plantId=2901812&query=Yuliren&filter=&fuzzy=false&nameType=all&dbs=wcsCmp) | Fruit | 15 |
| Gualou | *[Trichosanthes Kirilowii Maxim](https://old.tcmsp-e.com/tcmspsearch.php?qr=Trichosanthes%20Kirilowii%20Maxim&qsr=herb_en_name&token=3086ffb441b719d6cc0939a39a25d0a8)* | [Trichosanthes kirilowii Maxim.](https://mpns.science.kew.org/mpns-portal/plantDetail?plantId=2433223&query=Gualou&filter=&fuzzy=false&nameType=all&dbs=wcsCmp) | Fruit | 15 |
| Laifuzi | *Raphani Semen* | Raphanus raphanistrum subsp. sativus (L.) Domin | Fruit | 10 |
| Chaihu | *Radix Bupleuri* | Bupleurum falcatum L. | Root | 9 |
| Baishao | *[Paeoniae Radix Alba](https://old.tcmsp-e.com/tcmspsearch.php?qr=Paeoniae%20Radix%20Alba&qsr=herb_en_name&token=3086ffb441b719d6cc0939a39a25d0a8)* | [Paeonia lactiflora Pall.](https://mpns.science.kew.org/mpns-portal/plantDetail?plantId=519125&query=Baishao&filter=&fuzzy=false&nameType=all&dbs=wcs) | Root | 12 |
| Chenpi | *[Citrus Reticulata](https://old.tcmsp-e.com/tcmspsearch.php?qr=Citrus%20Reticulata&qsr=herb_en_name&token=3086ffb441b719d6cc0939a39a25d0a8)* | [Citrus × aurantium f. deliciosa (Ten.) M.Hiroe](https://mpns.science.kew.org/mpns-portal/plantDetail?plantId=2723971&query=Chenpi&filter=&fuzzy=false&nameType=all&dbs=wcsCmp) | Bark | 9 |
| Mangxiao | *Natrii Sulfas* | / | Rhizome | 3 |

**Table 2** Chemical characterization of bioactive compounds in LTD.

| **NO.** | **Name** | **Class** | **Formula** | **type** | **ppm** | **Intensity** |
| --- | --- | --- | --- | --- | --- | --- |
| 1 | Naringenin-7-O-rutinoside | Flavonoids | C_27_H_32_O_14_ | neg | 1.974005052 | 5545932175 |
| 2 | Neohesperidin | Flavonoids | C_28_H_34_O_15_ | pos | 0.04017997 | 2906934983 |
| 3 | Sinensetin | Flavonoids | C_20_H_20_O_7_ | pos | 0.334676492 | 2622257952 |
| 4 | 6-Demethoxytangeretin | Flavonoids | C_19_H_18_O_6_ | pos | 0.066143422 | 1643339098 |
| 5 | Isosinensetin | Flavonoids | C_20_H_20_O_7_ | pos | 0.303060594 | 1580202832 |
| 6 | Ferulaldehyde | Phenylpropanoids | C_10_H_10_O_3_ | pos | 2.089971697 | 1532423502 |
| 7 | Didymin | Flavonoids | C_28_H_34_O_14_ | pos | 0.444350053 | 1359213143 |
| 8 | 5-O-Demethylnobiletin | Flavonoids | C_20_H_20_O_8_ | pos | 0.428211042 | 992597923.2 |
| 9 | Gallocatechin | Flavonoids | C_15_H_14_O_7_ | neg | 0.90788157 | 793146776.6 |
| 10 | Katononic acid | Terpenoids | C_30_H_46_O_3_ | pos | 1.494671964 | 525139763.2 |
| 11 | Hesperetin | Flavonoids | C_16_H_14_O_6_ | neg | 0.086458733 | 429215732.7 |
| 12 | L-Tryptophan | Organoheterocyclic compounds | C_11_H_12_N_2_O_2_ | pos | 0.279174834 | 128094572 |
| 13 | Naringin | Flavonoids | C_27_H_32_O_14_ | neg | 0.880426586 | 86363226.43 |
| 14 | Baicalin | Flavonoids | C_21_H_18_O_11_ | pos | 1.488468822 | 84119380.26 |
| 15 | Arginine | Amino acid derivatives | C_6_H_14_N_4_O_2_ | pos | 1.477657544 | 56472413.62 |
| 16 | Kaempferide | Flavonoids | C_16_H_12_O_6_ | pos | 1.8908847 | 50090428.14 |
| 17 | Naringenin | Flavonoids | C_15_H_12_O_5_ | pos | 0.719204713 | 46468635.5 |
| 18 | Paeoniflorin | Terpenoids | C_23_H_28_O_11_ | pos | 0.99142093 | 42442676.56 |
| 19 | Liquoric acid | prenol lipids | C_30_H_44_O_5_ | pos | 1.371245562 | 33827304.11 |
| 20 | Saikosaponin D | Terpenoids | C_42_H_68_O_13_ | pos | 0.791614188 | 19661050.81 |

Analysis of the components of LTD by UHPLC-QTOF-MS. The total ion chromatograms (TIC) of QCHS. (A) The positive mode and (B) the negative mode.

A


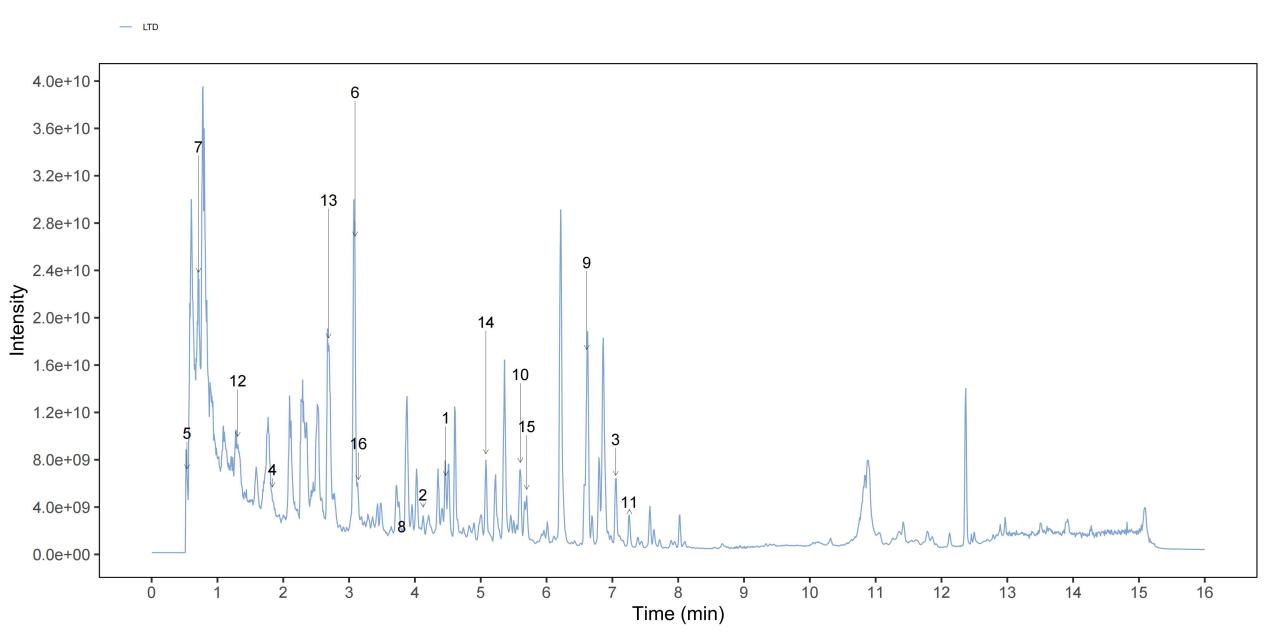


B


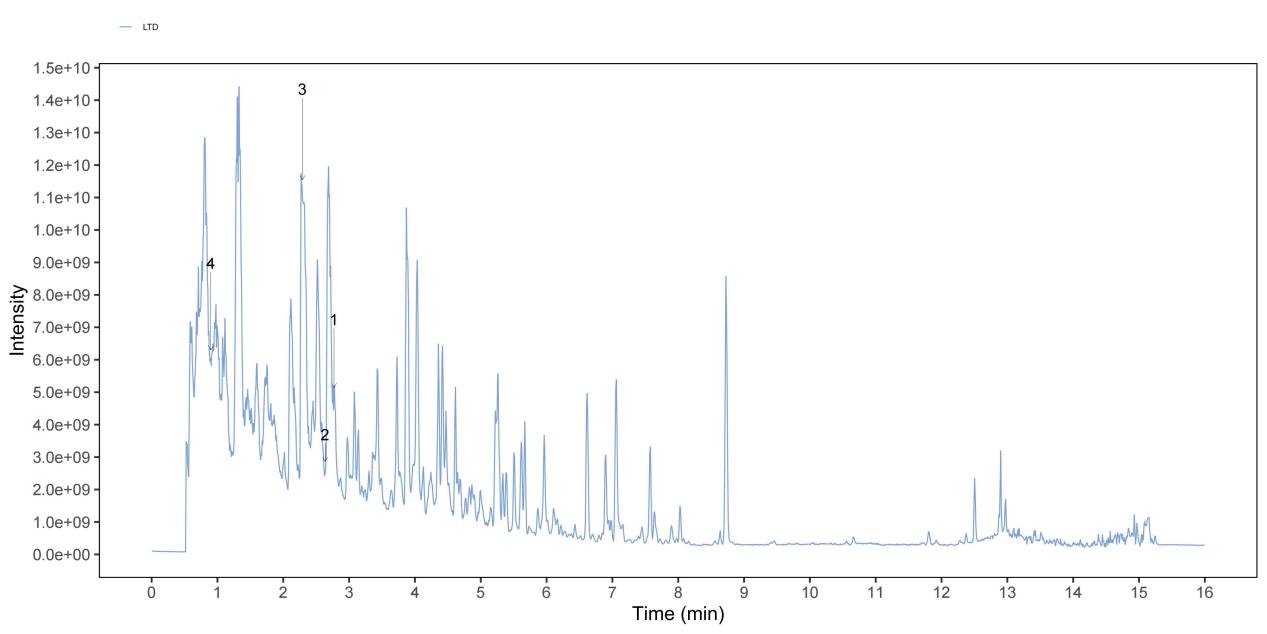


**Table 3 Correlation analysis of differential Intestinal flora with 5-HT concentration**

| Item | Target 1 | Target 2 | *R^2^* | *P* |
| --- | --- | --- | --- | --- |
| Intestinal flora | Lachnospiraceae_NK4A136 | 5-HT | 0.713 | 0.009 |
|  | Firmicutes |  | 0.738 | 0.006 |
|  | Clostridiales |  | 0.748 | 0.005 |
|  | Haemophilus |  | 0.847 | 0.001 |
|  | Christensenellaceae_R-7 |  | -0.916 | 0.000 |
|  | Alloprevotella |  | -0.697 | 0.012 |
|  | Dorea |  | -0.702 | 0.011 |
|  | Parabacteroides |  | -0.811 | 0.001 |
|  | Coprococcus |  | -0.942 | 0.000 |
|  | Quinella |  | 0.806 | 0.002 |
|  | Eubacterium_oxidoreducens |  | 0.776 | 0.003 |
|  | Paramuribaculum |  | 0.694 | 0.012 |
|  | Corynebacterium |  | 0.723 | 0.008 |
|  | Anaerotruncus |  | 0.716 | 0.009 |
|  | Candidatus_Saccharimonas |  | 0.703 | 0.011 |
|  | Bifidobacterium |  | -0.780 | 0.003 |
|  | Marvinbryantia |  | -0.776 | 0.003 |
|  | Phascolarctobacterium |  | -0.725 | 0.008 |
|  | Collinsella |  | -0.902 | 0.000 |
|  | Faecalibacterium |  | -0.877 | 0.000 |

**Table 4 Correlation analysis of differential SCFAs with 5-HT concentration**

| Item | Target 1 | Target 2 | *R^2^* | *P* |
| --- | --- | --- | --- | --- |
| SCFAs | Acetic acid | 5-HT | 0.544 | 0.068 |
|  | Propionic acid |  | 0.500 | 0.098 |
|  | Isobutyric acid |  | 0.655 | 0.021 |
|  | Butyric acid |  | 0.636 | 0.026 |
|  | Valeric acid |  | 0.690 | 0.013 |
|  | Hexanoic acid |  | 0.783 | 0.003 |
|  | Heptanoic acid |  | 0.818 | 0.001 |
|  | Decanoic acid |  | 0.749 | 0.005 |

**Supplementary Figure**


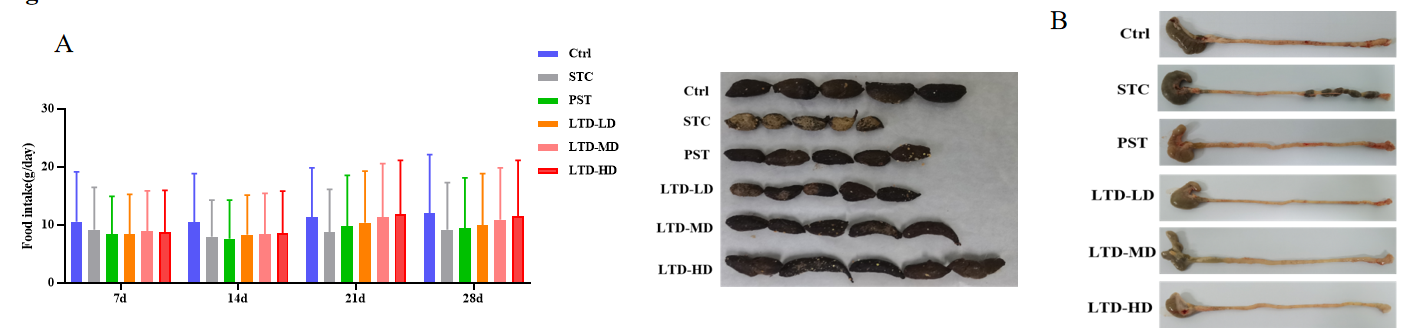


**Figure 1.** Effects of LTD on General Behavioral Changes in STC Rats with Qi Stagnation Pattern. (A)Food intake, comparison of fecal traits ,and **(B)** residual colon feces in different groups of rats.


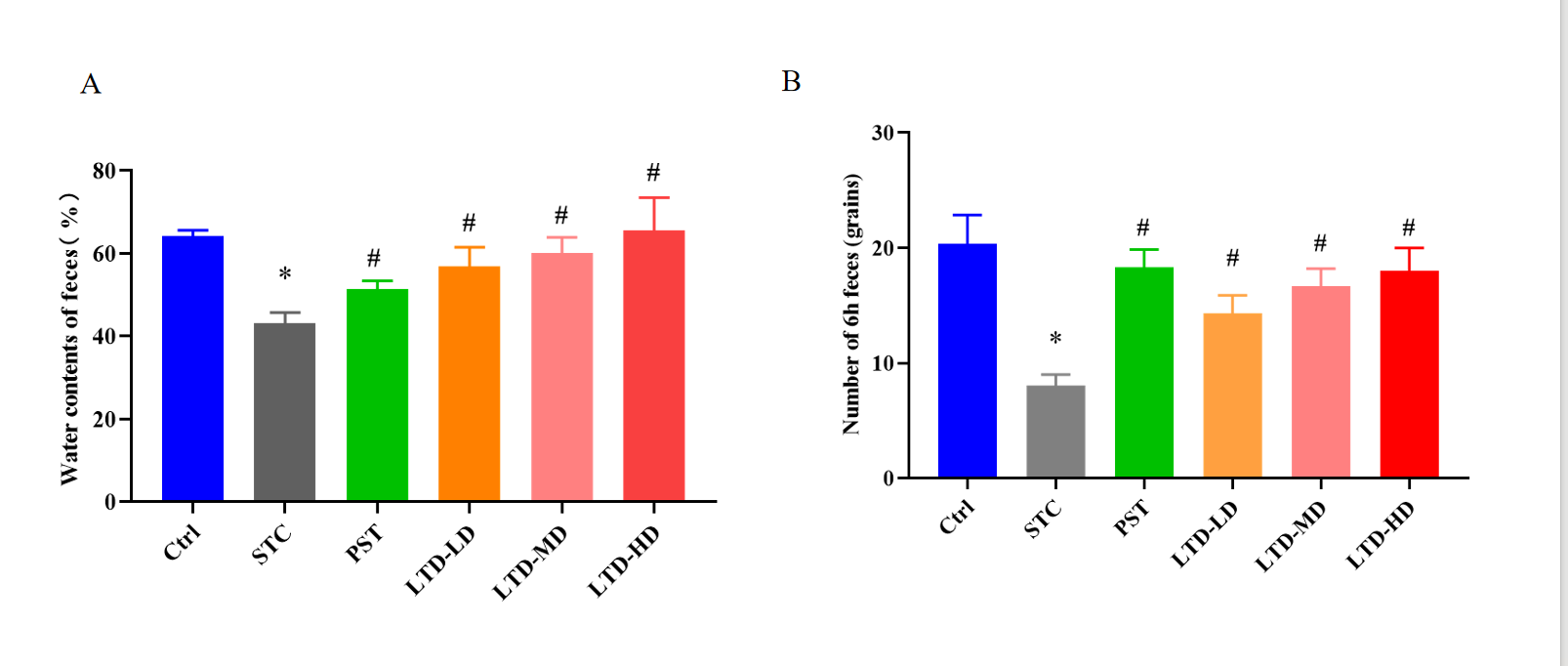


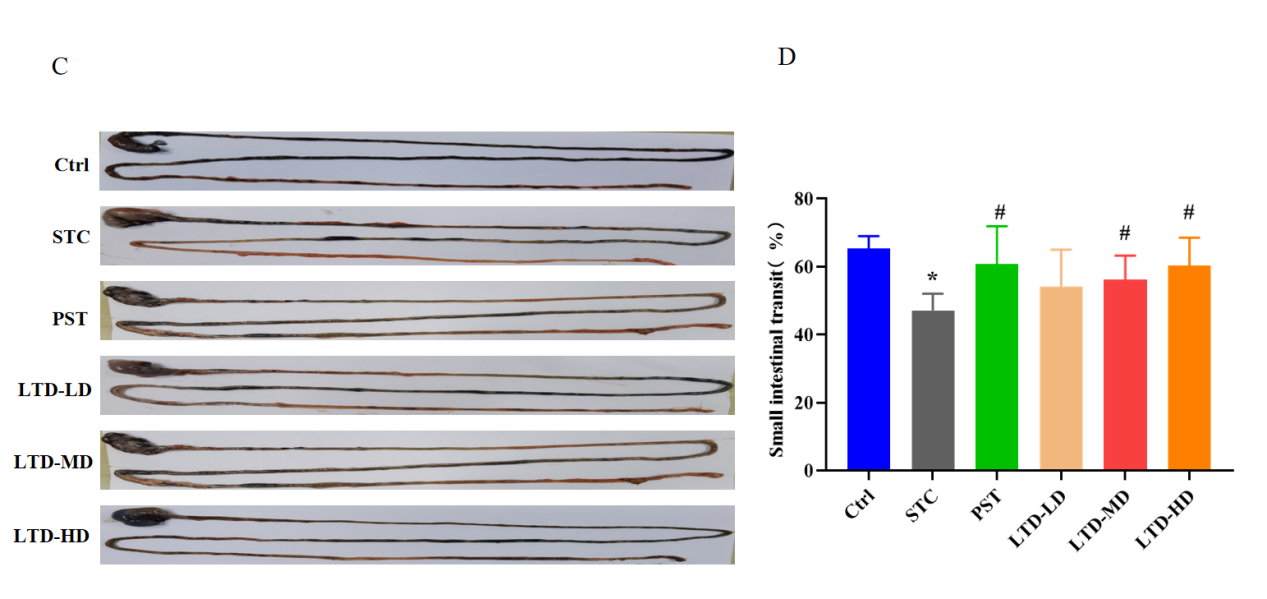


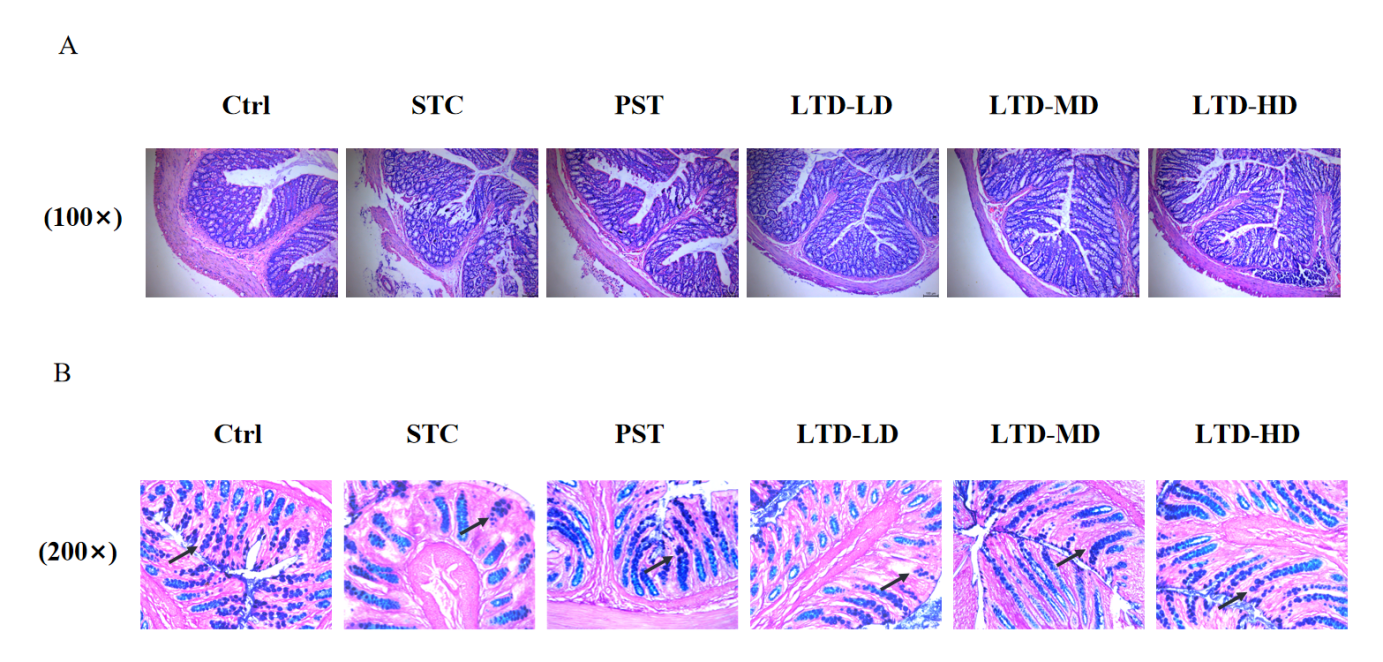
**Figure 2.** Improved defecation and intestinal transit in loperamide-induced STC rats by LTD. **(A)** Fecal water content, **(B)** 6h number of feces, **(C)** intestinal propelling movement of carbon ink and **(D)** Intestinal propulsive rate. **p* < 0.05 vs. Normal, ^#^ *p* < 0.05 vs. STC.

**Figure 3.** LTD improved the histopathology and goblet cells count of colon in STC rats with Qi Stagnation Pattern. **(A)** HE staining results of colon tissue, **(B)** AB-PAS staining results of colon tissue.


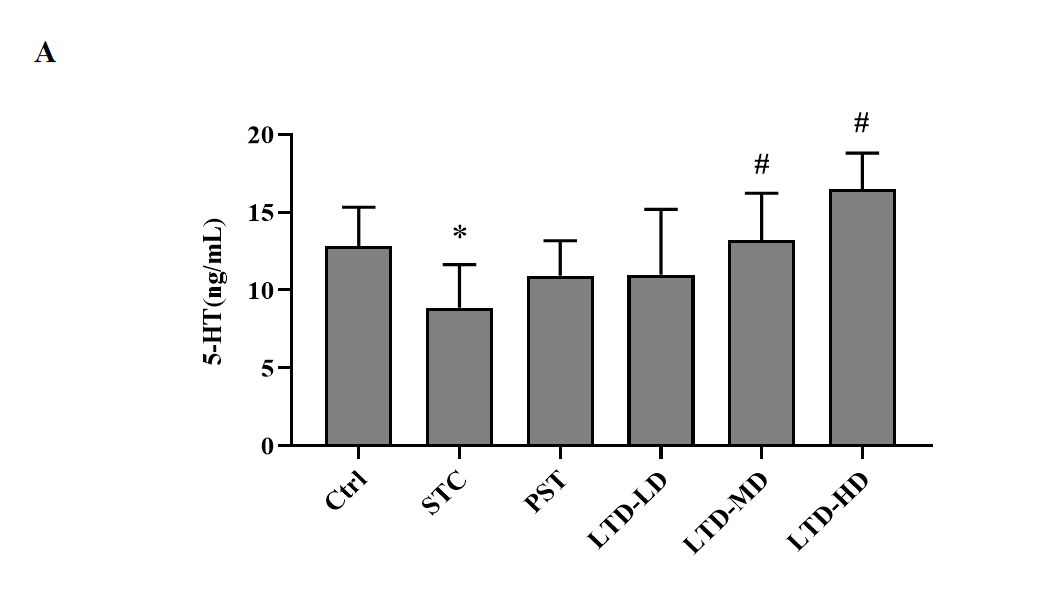


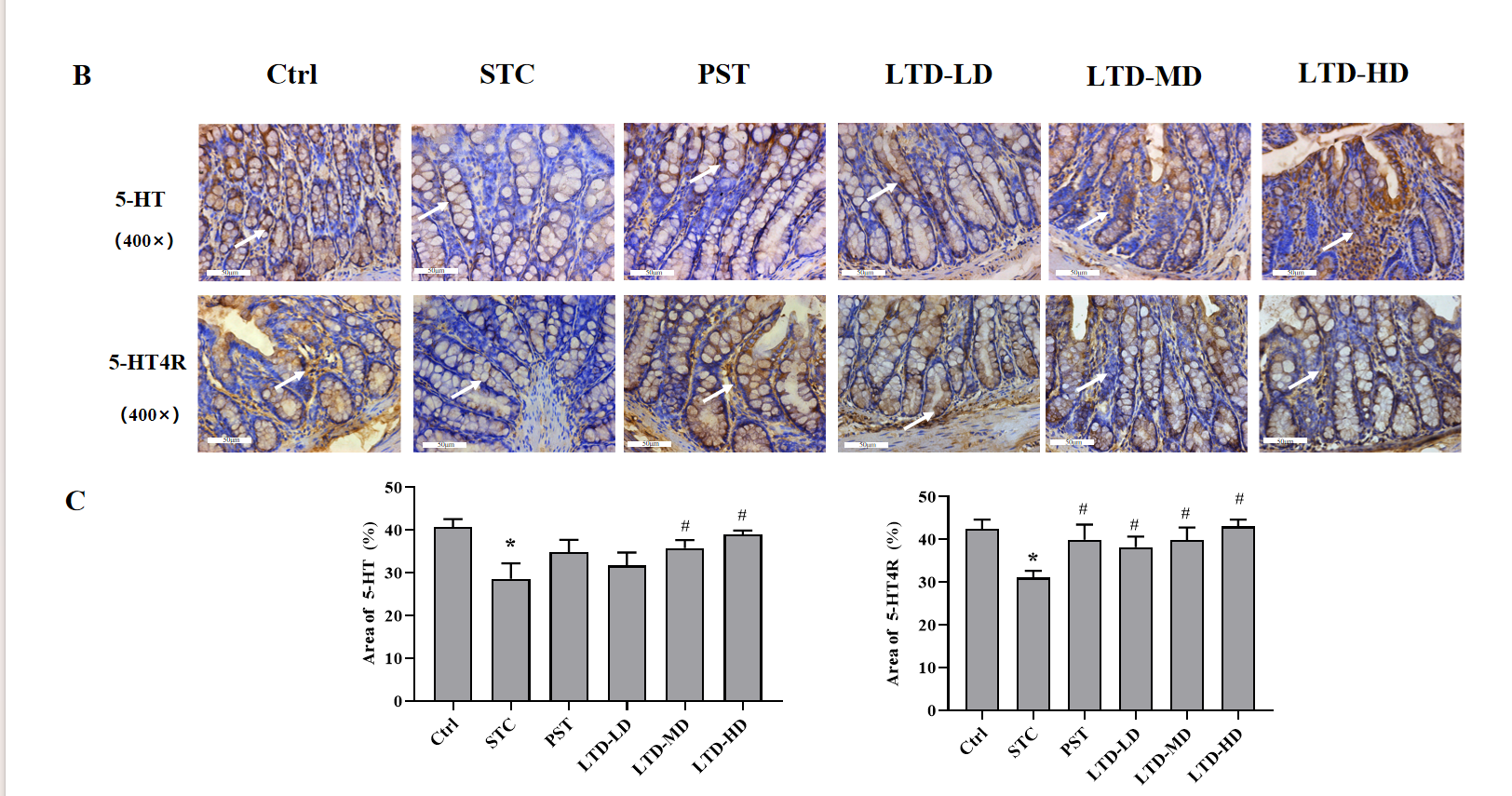


**Figure 4.** LTD promoted the secretion of 5-HT and activated its receptor pathway in the STC rats with Qi Stagnation Pattern. **(A)** ELISA results of 5-HT in serum. **(B-C)** Immunohistochemical results of 5-HT and 5-HT4R in colon tissue. **p* < 0.05 vs. Normal, ^#^ *p* < 0.05 vs. STC.


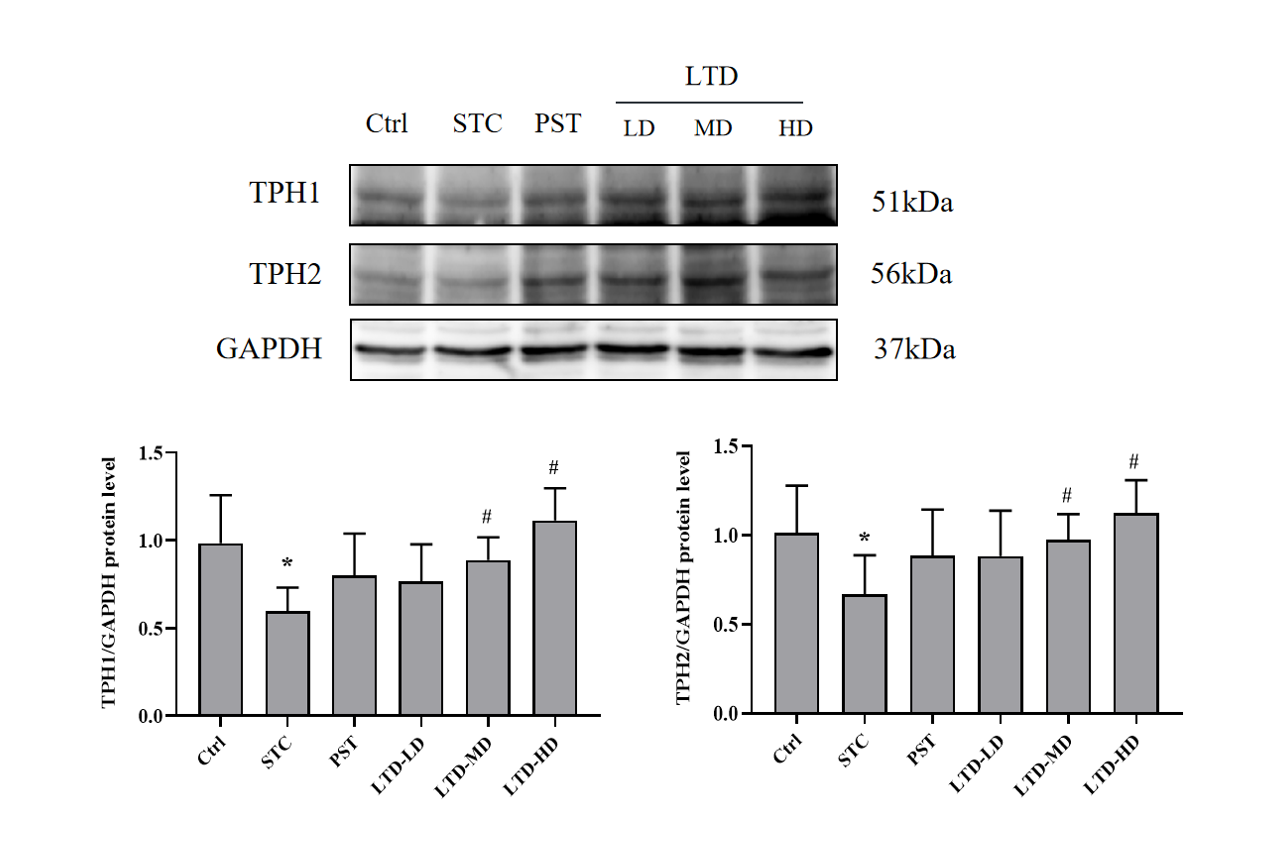


**Figure 5.** LTD upregulated TPH1 and TPH2 protein levels in STC rats with Qi Stagnation Pattern. GAPDH was used as the internal standard. **p* < 0.05 vs. Normal, ^#^ *p* < 0.05 vs. STC.


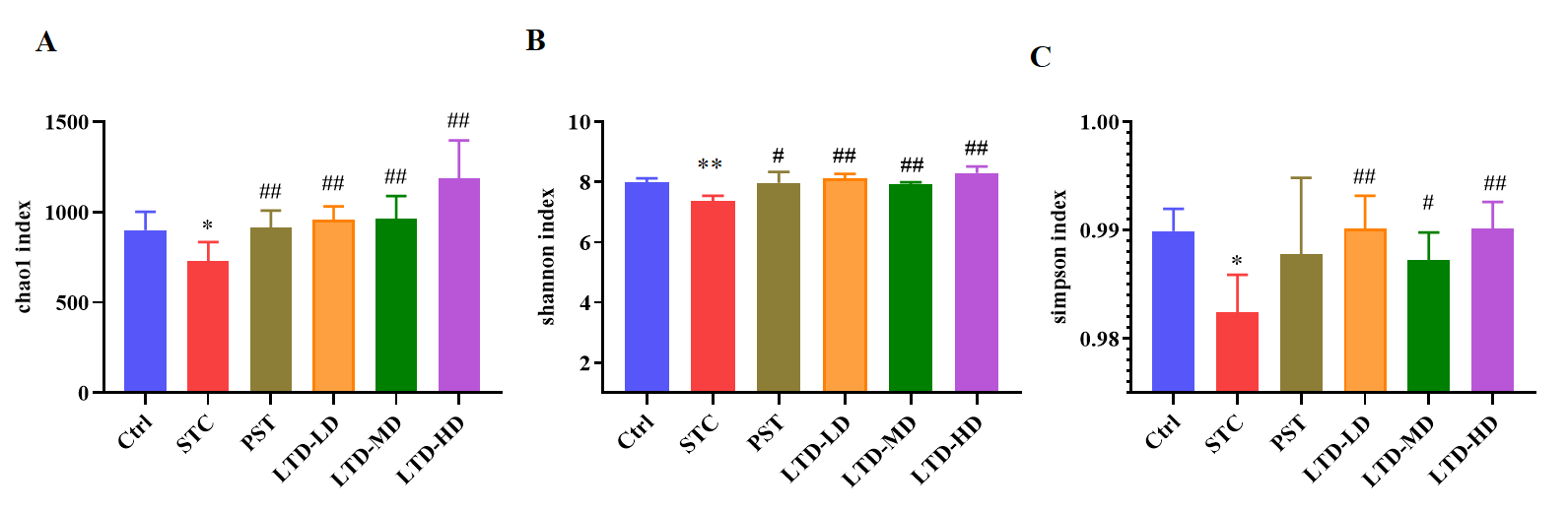


**Figure 6.** The alpha diversity of microbiomes identified in STC rats treated with LTD. The microbial abundances within the group were compared based on the chao1, shannon and simpson separately. **p* < 0.05 ,***p* < 0.01 vs. Normal, ^#^ *p* < 0.05, ^##^*p* < 0.01 vs. STC.


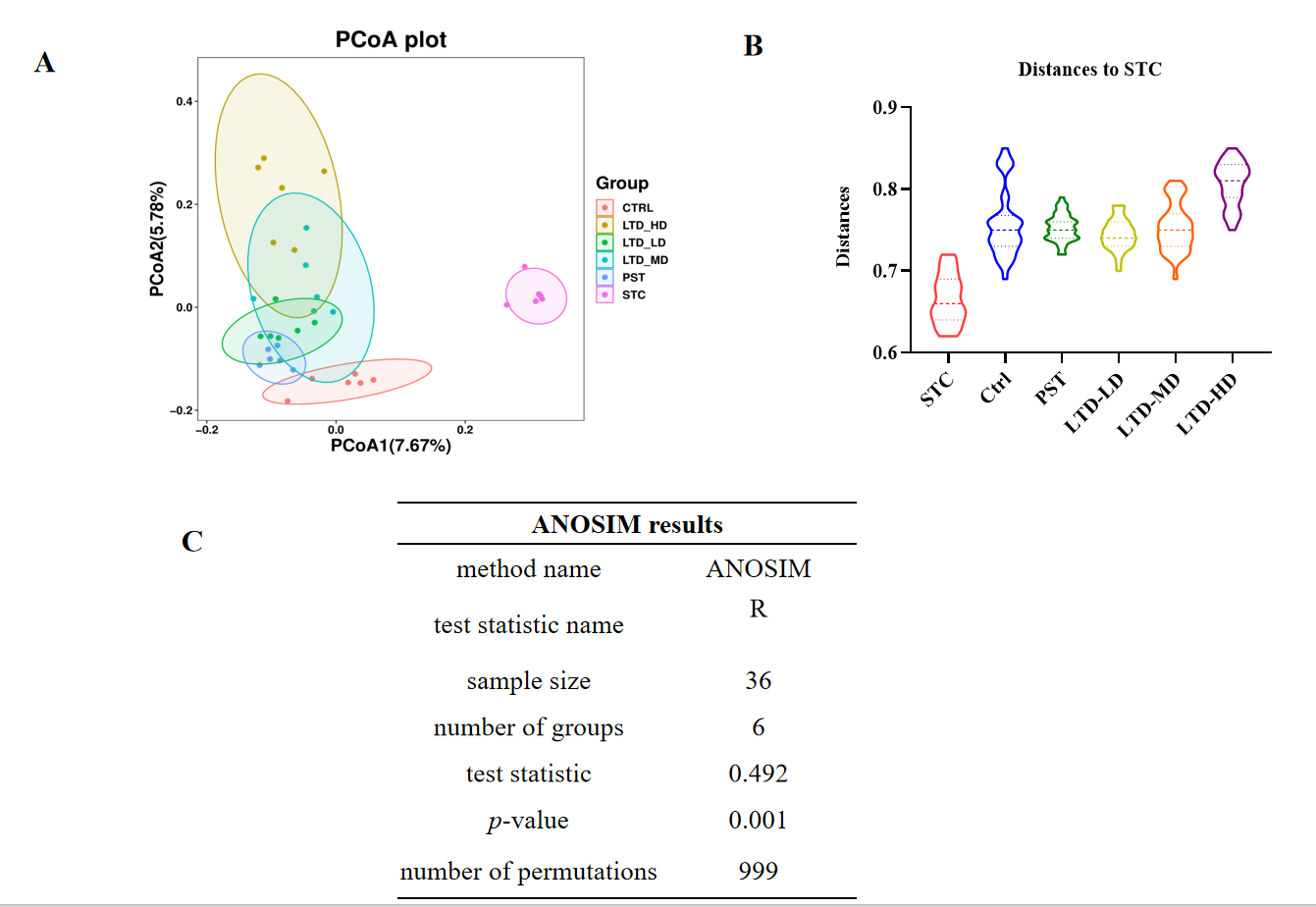


**Figure 7.** The beta diversity of microbiomes characterized in the STC model rats with LTD treatment. **(A)** PCoA analysis based on Unweighted UniFrac, **(B)** distances to STC,and **(C)** ANOSIM method results.


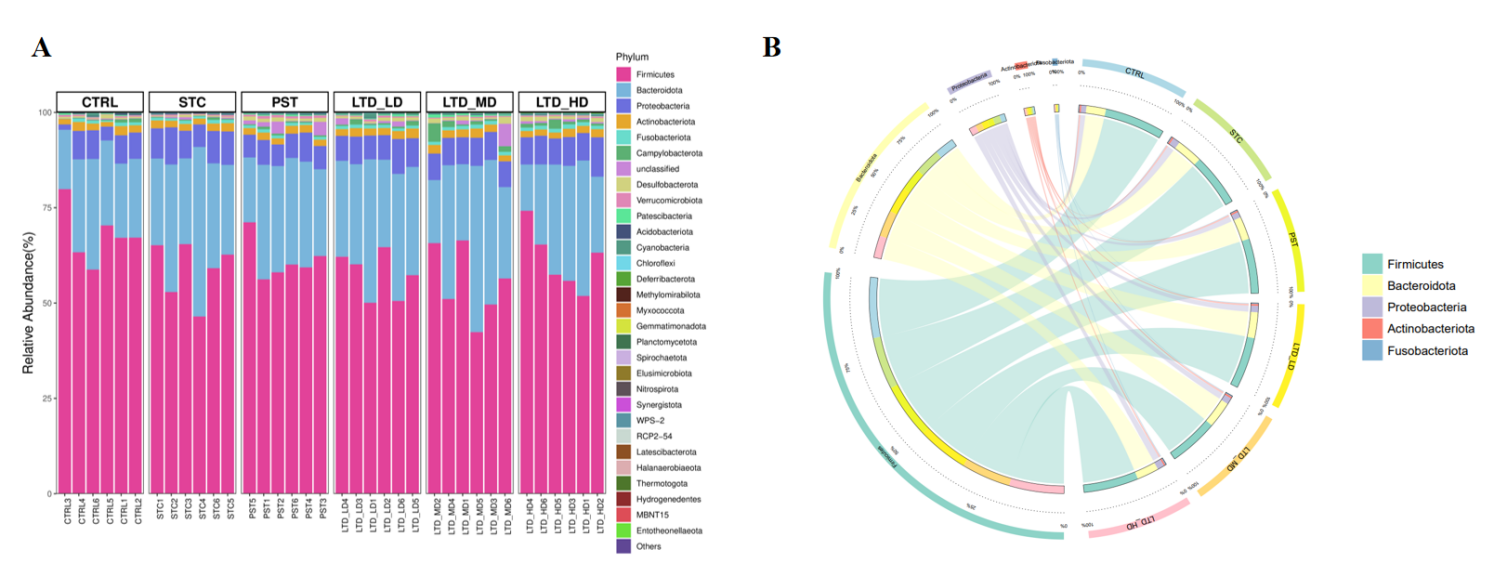


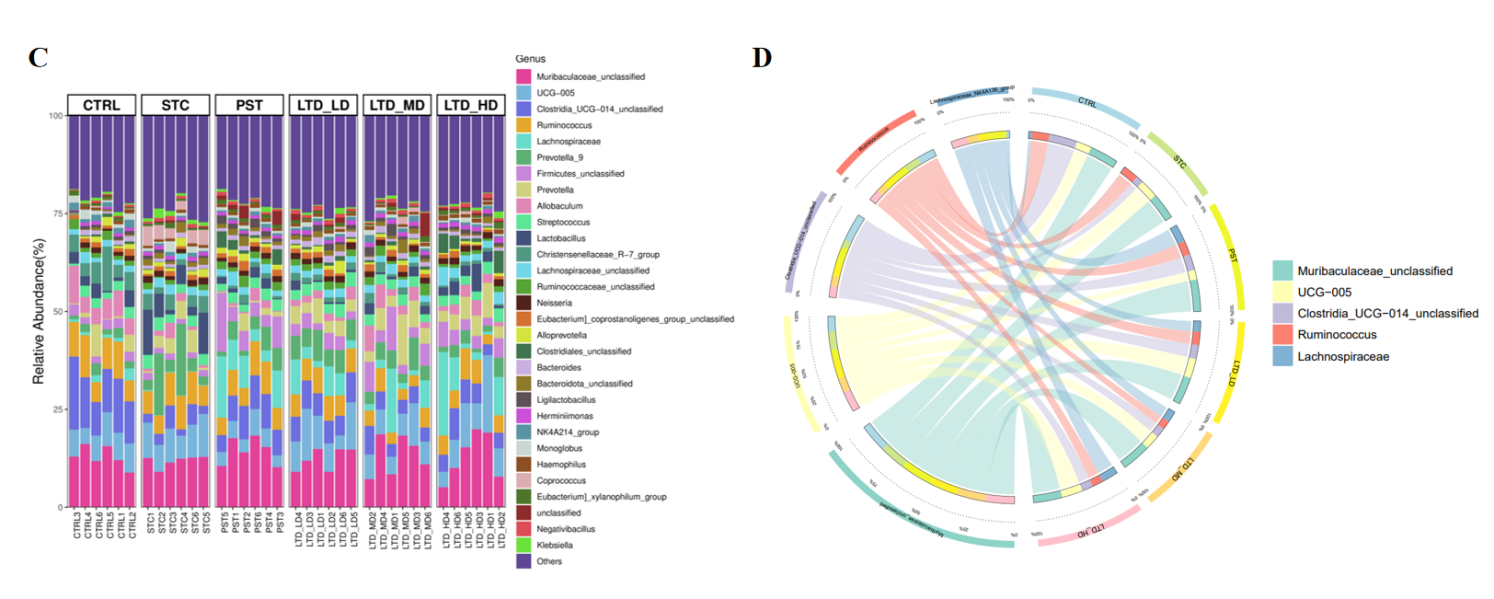


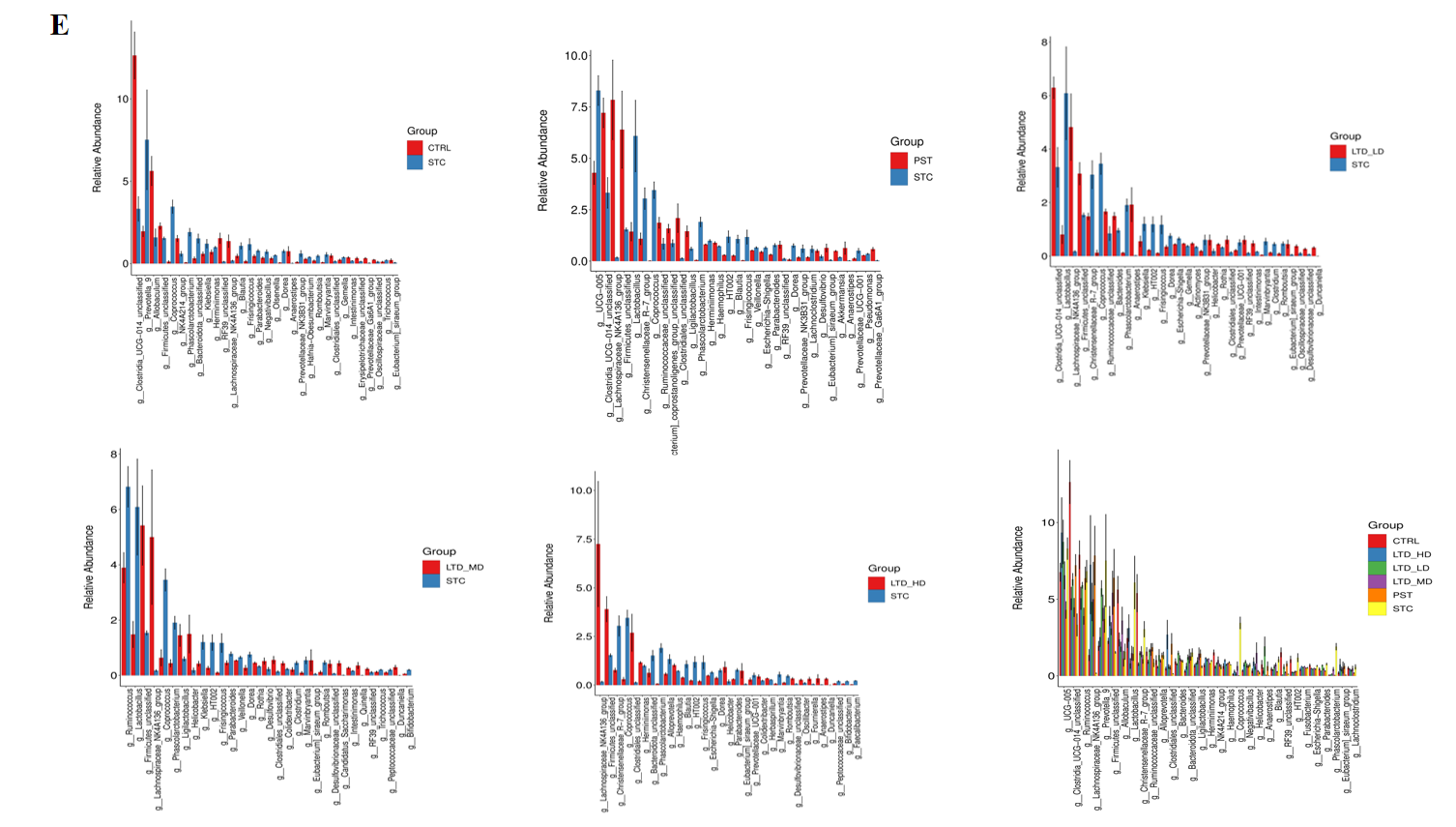


**Figure 8.** Microbiome alterations at various taxonomic levels in STC rats after LTD treatment. The microbiome profile alterations among the six mouse groups were compared at the levels of phylum **(A,B)** and genus **(C,D)**. **(E)**Comparison of intestinal flora between groups at the genus level.


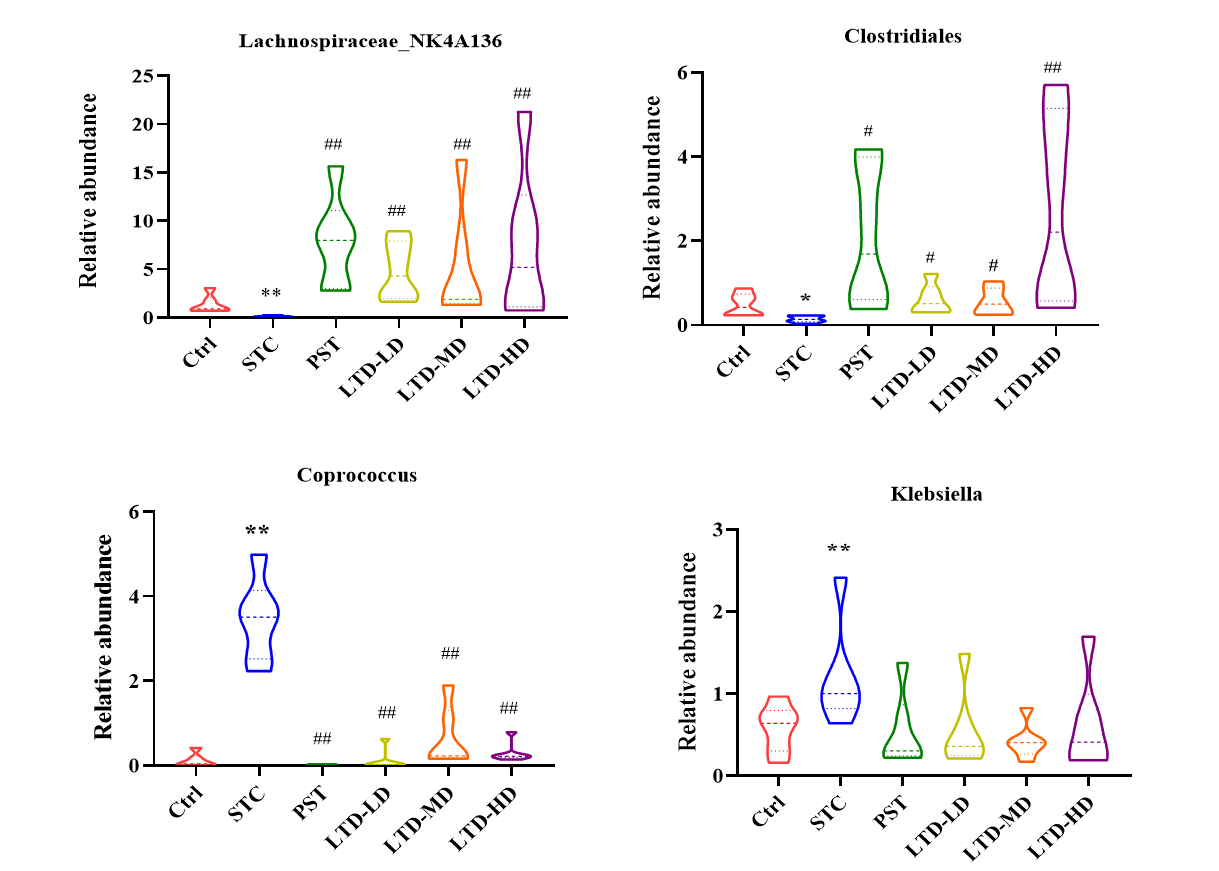


**Figure 9.** Relative abundances of Lachnospiraceae_NK4A136, Clostridiales, Coprococcus and Klebsiella in fecal samples collected from STCrats treated with LTD. **p* < 0.05 ,***p* < 0.01 vs. Normal, ^#^ *p* < 0.05, ^##^*p* < 0.01 vs. STC.


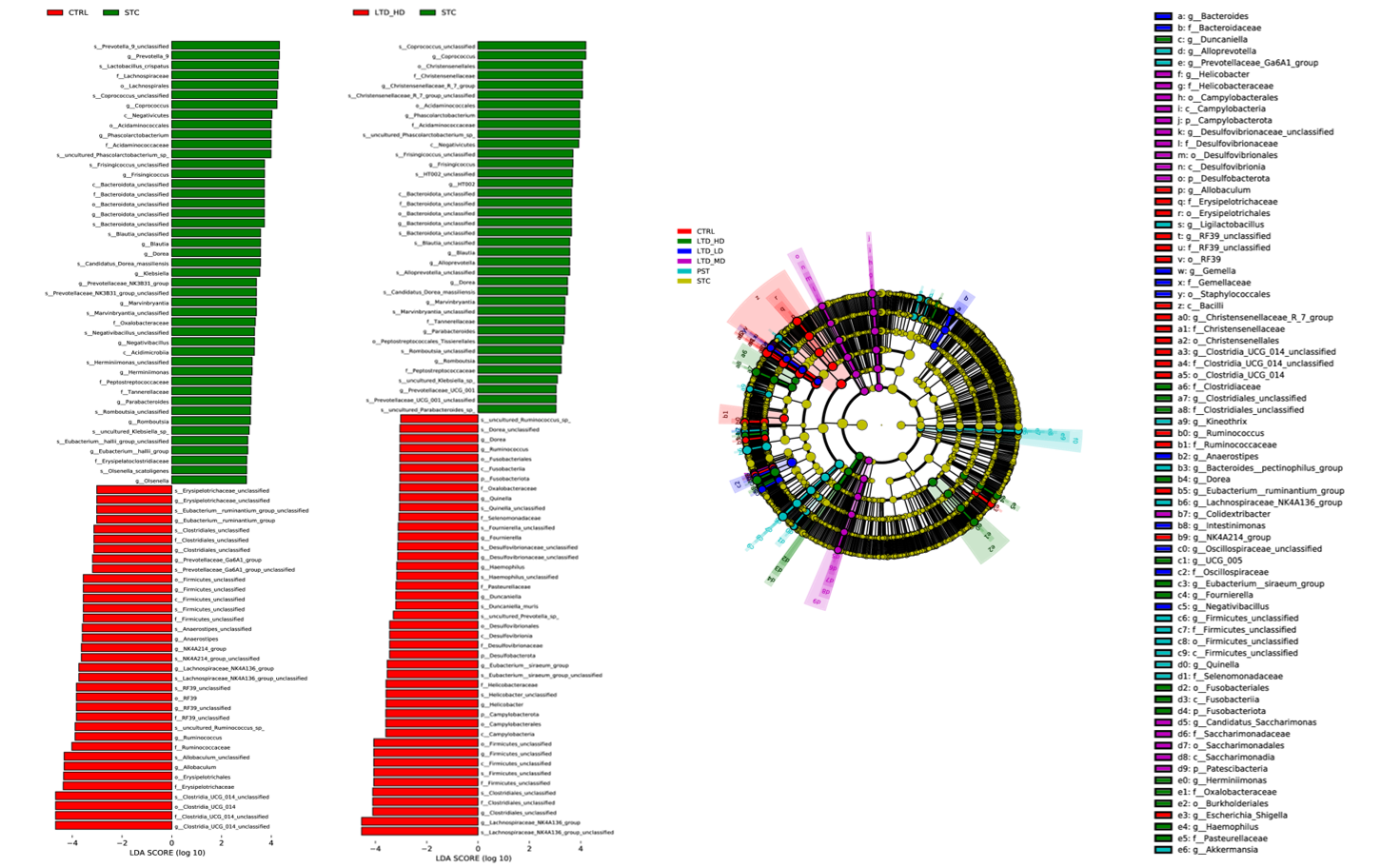


**Figure 10.** Taxonomic cladogram of major differential microbial species in different groups of rats through LEfSe analysis. Biomarker taxa are highlighted with colored circles and shaded areas. Each circle’s diameter reflects the abundance of those taxa in the community.


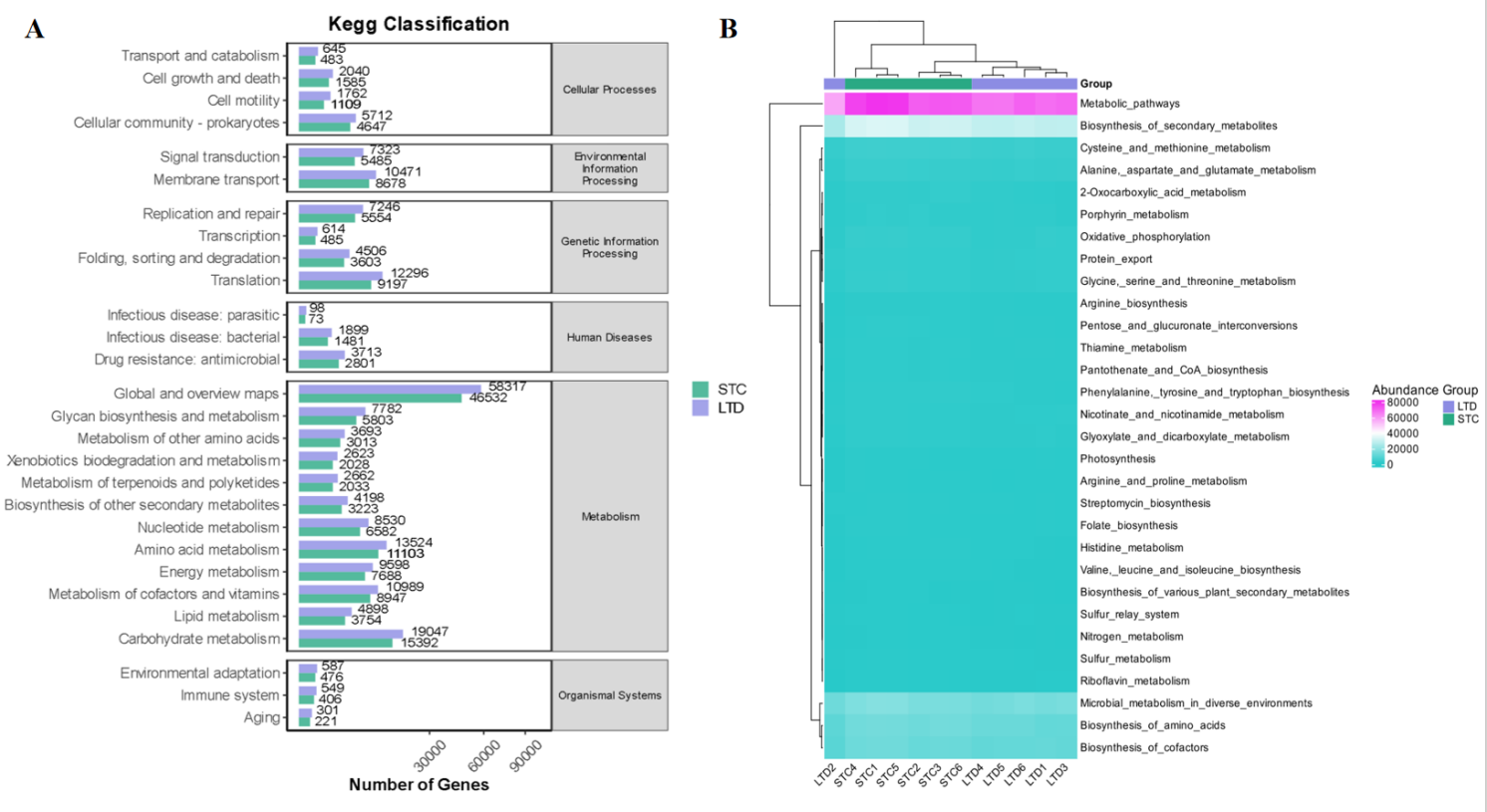


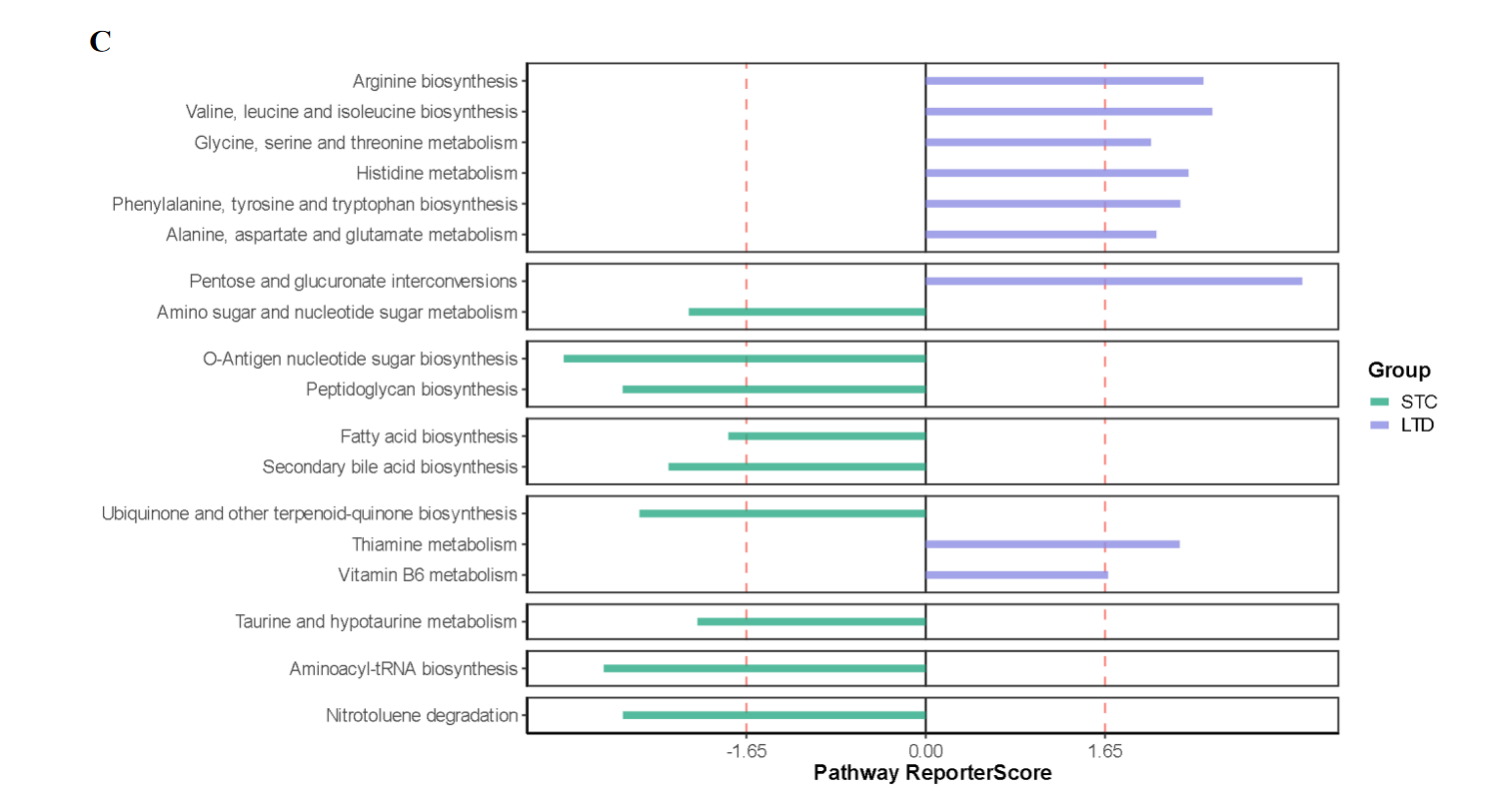


**Figure 11.** Functional prediction analysis（between STC and LTD group）: **(A)**  KEGG level first function prediction. **(B)** KEGG secondary function prediction. **(C)** KEGG enrichment analysis.


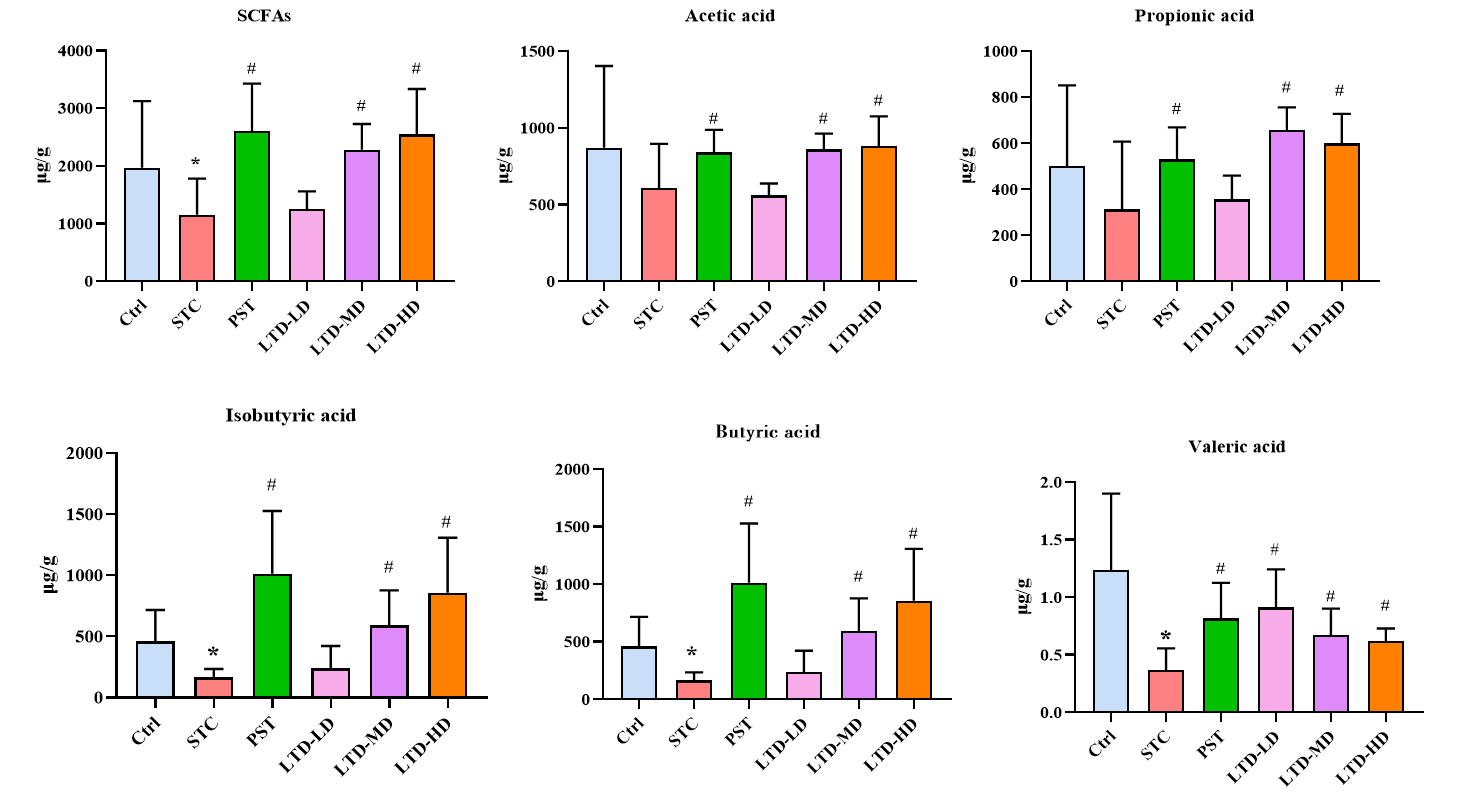


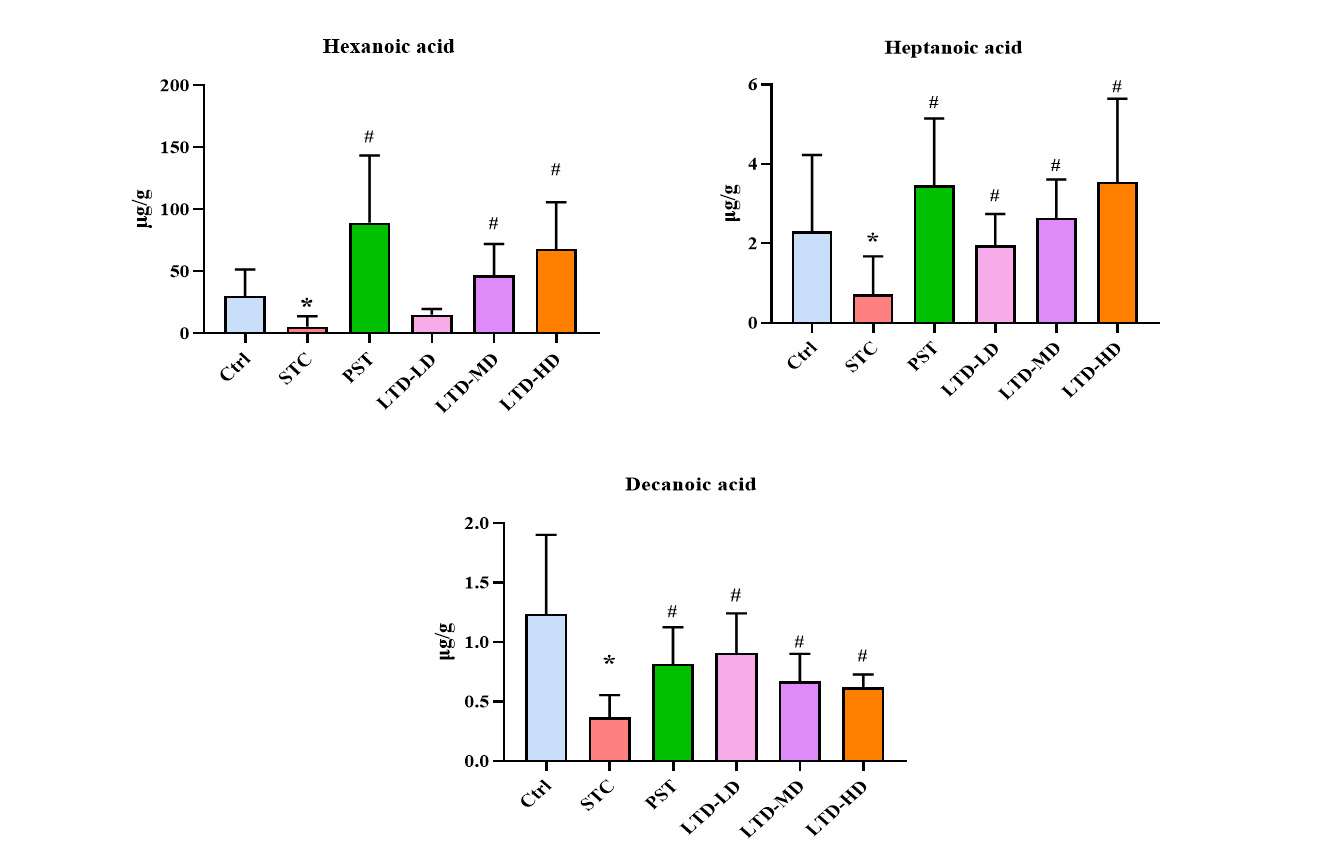


**Figure 12.** Effects of LTD on short-chain fatty acids (SCFAs) in colonic contents of the STC rats with Qi Stagnation Pattern. **p* < 0.05 vs. Normal, ^#^ *p* < 0.05 vs. STC.


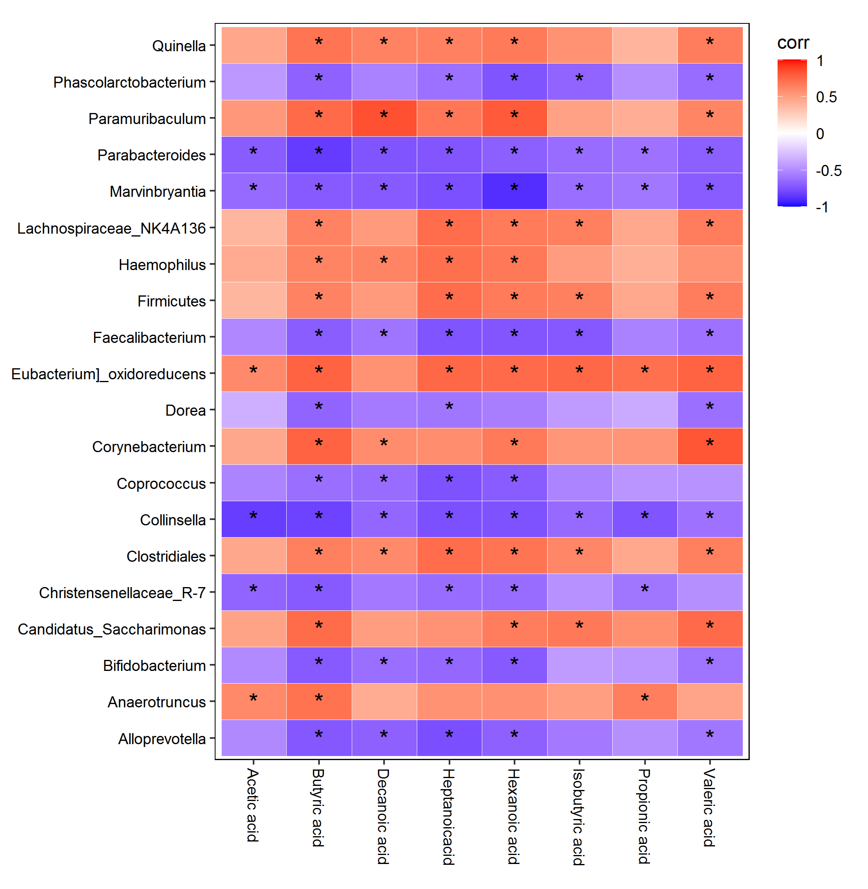


**Figure 13.** Spearman Correlation Analysis Between the SCFAs, 5-HT and the Colonic Microflora
